# Genetic variability, management, and conservation implications of the critically endangered Brazilian pitviper *Bothrops insularis*

**DOI:** 10.1002/ece3.6838

**Published:** 2020-10-03

**Authors:** Igor Salles‐Oliveira, Taís Machado, Karina Rodrigues da Silva Banci, Selma M. Almeida‐Santos, Maria José de J. Silva

**Affiliations:** ^1^ Laboratório de Ecologia e Evolução Instituto Butantan São Paulo Brazil

**Keywords:** genetic variability, in situ and ex situ conservation, population structure, Serpentes, SSR

## Abstract

Information on demographic, genetic, and environmental parameters of wild and captive animal populations has proven to be crucial to conservation programs and strategies. Genetic approaches in conservation programs of Brazilian snakes remain scarce despite their importance for critically endangered species, such as *Bothrops insularis,* the golden lancehead, which is endemic to Ilha da Queimada Grande, coast of São Paulo State, Brazil. This study aims to (a) characterize the genetic diversity of ex situ and in situ populations of *B. insularis* using heterologous microsatellites; (b) investigate genetic structure among and within these populations; and (c) provide data for the conservation program of the species. Twelve informative microsatellites obtained from three species of the *B. neuwiedi* group were used to access genetic diversity indexes of ex situ and in situ populations. Low‐to‐medium genetic diversity parameters were found. Both populations showed low—albeit significant—values of system of mating inbreeding coefficient, whereas only the in situ population showed a significant value of pedigree inbreeding coefficient. Significant values of genetic differentiation indexes suggest a small differentiation between the two populations. Discriminant analysis of principal components (DAPC) recovered five clusters. No geographic relationship was found in the island, suggesting the occurrence of gene flow. Also, our data allowed the establishment of six preferential breeding couples, aiming to minimize inbreeding and elucidate uncertain parental relationships in the captive population. In a conservation perspective, continuous monitoring of both populations is demanded: it involves the incorporation of new individuals from the island into the captive population to avoid inbreeding and to achieve the recommended allelic similarity between the two populations. At last, we recommend that the genetic data support researches as a base to maintain a viable and healthy captive population, highly genetically similar to the in situ one, which is crucial for considering a reintroduction process into the island.

## Graphical abstract

This is the first report focusing on the conservation genetics of *Bothrops insularis*, an endemic critically endangered Brazilian snake. We recovered low (allelic content) and medium (heterozygosity) genetic diversity indexes, significant inbreeding coefficient for the in situ population and a slight genetic structure within and between populations. Based on these results, we suggest some strategies that aim to reduce extinction risks, minimize genetic divergence, and genetic loss.

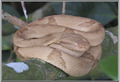

## 1 INTRODUCTION

Genetic diversity is one of the three classes of biodiversity recognized as global conservation priorities and plays a decisive role in conservation efforts (AZA, 2020; IUCN – International Union for Conservation of Nature, 2019; Sodhi & Ehrlich, 2010). Genetic diversity data on wild and captive populations have shown to be useful for evaluating the consequences of fragmentation and habitat loss, elucidating gene flow and population genetic structure, and defining important evolutionary areas (see Arruda et al., 2017; Gallego‐Gárcia et al, 2018; Madsen et al., 2000; McAlileyet al., 2016; Michaelidis et al., 2015; Monzón‐Argüello et al., 2015; Wallis, 2019). However, a recent review on 30 years of conservation genetics in New Zealand, Wallis (2019) highlighted that genetic approaches have some limitations related to the definition of minimum viable populations, eco‐sourcing, inference of gene flow, and species boundaries. To address these limitations, genetic approaches should grief with other knowledges (for example, demography and reproductive traits) in an integrative conservation effort (Allendorf et al., 2013).

Both ex situ and in situ conservation programs focus on retaining genetic diversity for a minimum period—usually 100 years (Frankham et al., 2008). However, demographic variations associated with captive adaptation, anthropogenic impacts, or the occurrence of inbreeding lead to a decrease in genetic diversity. In several cases, this may carry populations toward an extinction vortex and imminent extinction, as occurred to the isle royal wolves (*Canis lupus*) (Frankham, 2005; Frankham, 2010; Hedrick et al., 2019; West et al., 2018). Based on this, the management of wild and captive populations has focused on the maintenance of a viable population and its genetic diversity through the avoidance of inbreeding and captive adaptation, and a continuous monitoring of demographic events and genetic diversity (IUCN, 2002; Shafer et al., 2015).

Brazil is considered as a megadiverse South American country due to its high levels of species richness and endemism, with biodiversity hotspot regions (e.g., Cerrado, Rainforest; ICMBio/MMA, 2018a). The last census of Brazilian fauna identified 34 snake species classified as vulnerable, endangered, or critically endangered (ICMBio/MMA, 2018b), most of which are incorporated into national conservation plans. Other than improvement of Brazilian snakes conservation (Navega‐Gonçalves & Porto, 2016), only one study focuses on genetic data using Random Amplified Polymorphic DNA (RAPD) in *Bothrops moojeni* populations (see Dutra et al., 2008).

The genus *Bothrops* is a neotropical pitviper group widely distributed in Brazil, which plays an important ecologic role, apart from its utility in medicine (Campbell & Lamar, 2004). This genus encompasses 45 species (Uetz, Freed, & Hošek, 2020), 10 out of which are described as endemic and/or threatened or near‐threatened (Navega‐Gonçalves & Porto, 2016; Rodrigues, 2005). The golden lancehead, *Bothrops insularis*, is an endemic and critically endangered species from Ilha da Queimada Grande (Marques et al., 2002), a small island (0.43 km^2^) located 33 km off the coast of São Paulo State (24°30′S and 43°42′O) that is part of a conservation unit classified as “Área de Relevante Interesse Ecológico (ARIE) das Ilhas da Queimada Pequena e Queimada Grande” (Brasil, 1985).

Even though the access in the island is restricted to the marine and authorized scientific researchers, natural and deliberative bushfires, and anthropogenic actions such as capture of specimens and biopiracy have already been reported (Duarte et al., 1995; Guimarães et al., 2014; Martins et al., 2008). These threats associated with restrict geographic distribution and evidence of populational decline lead this species to be classified as critically endangered (according to the parameters B1ab (iii) + 2ab (iii)) by the International Union for Conservation of Nature (IUCN, 2019) and the Red List of Brazilian Fauna (ICMBio/MMA, 2018b).

The majority of the studies with *B. insularis* focused predominantly on ecology (Guimarães et al., 2014; Marques et al., 2012; Martins et al., 2008), reproduction (Amorim et al., 2019; Marques et al., 2013; Silva et al., 2015), and phylogeography (Grazziotin et al., 2006). Populational genetics and molecular parameters of the species, however, remain scarce, even though accumulation of genetic information and maintenance of genetically viable ex situ populations of the species were a component of the aims included in a national plan specific for insular herpetofauna (Bataus et al., 2011), which may allow the design of future reintroduction plans.

Based on this brief historic, due to this species vulnerability, ex situ conservation programs have been designed in Brazilian scientific centers, such as the Instituto Butantan. The ex situ population housed at the Laboratório de Ecologia e Evolução, Instituto Butantan, was established in 2009/2010 with 20 founders (IBAMA no. 25.650‐1), aiming to develop a healthy population which could be used for either scientific researches or future reintroduction (Kasperoviczus & Almeida‐Santos, 2012). Up to now, breeding couples were designed based on the sperm health and viability for male's selection (Silva et al., 2015), and x‐ray analysis to select females in vitellogenesis. Although mating was reported, no study book was maintained for this population.

In this context, knowing that the access of genetic data of wild and captive populations is crucial for conservation programs, our study aims to: (a) characterize the genetic diversity of ex situ and in situ populations of *B. insularis* using heterologous microsatellites; (b) investigate the existence of genetic structures among and within populations; and (c) provide data for the conservation program of the species.

## 2 MATERIALS AND METHODS

### 2.1 Sample collection

**Figure 1:**
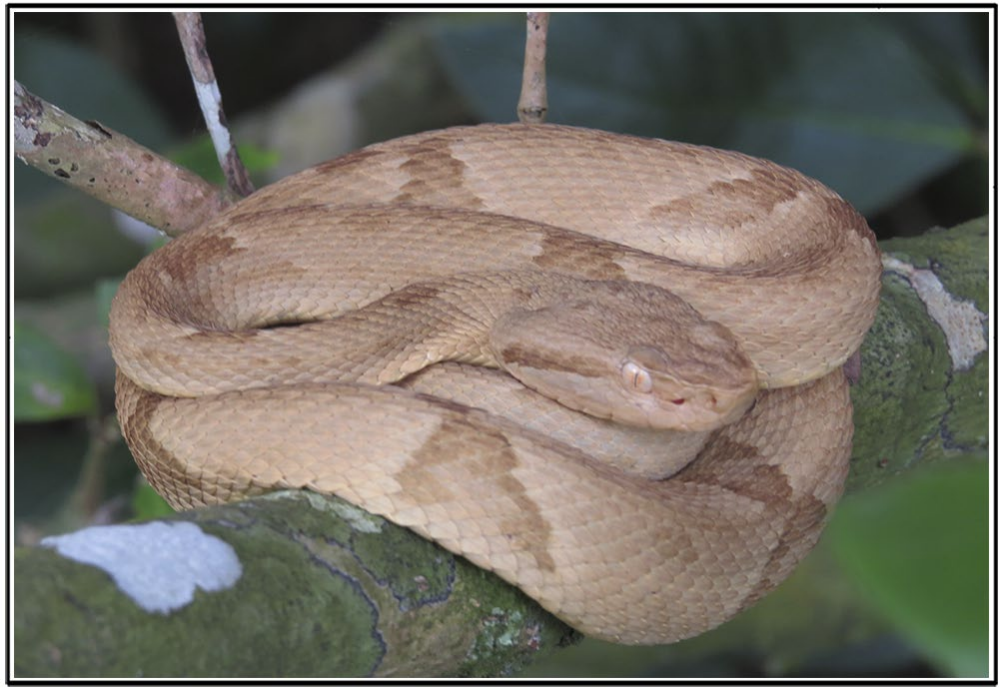
*Bothrops insularis* from Ilha da Queimada Grande, São Paulo State, Brazil. Source: Karina Banci

**Figure 2:**
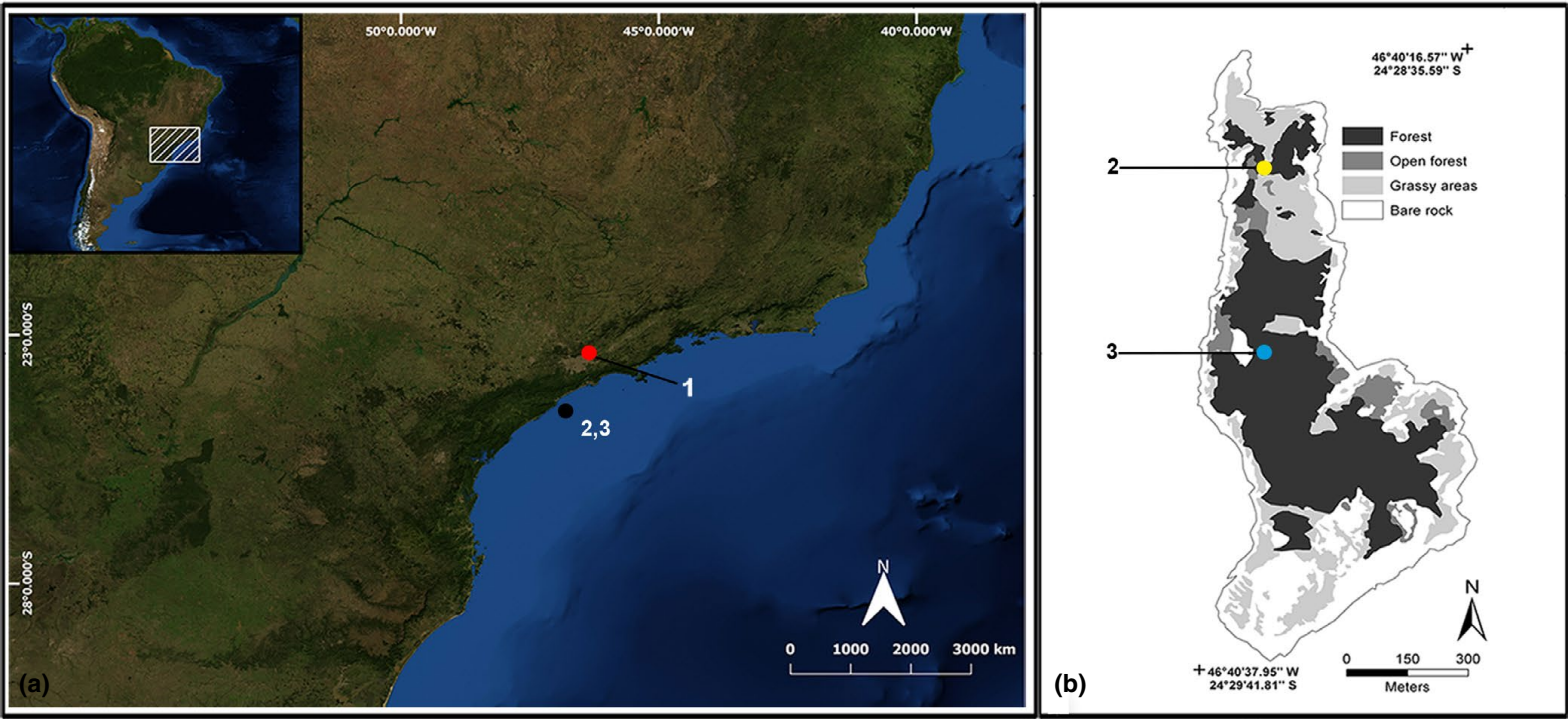
Sampling locations of the *Bothrops insularis* populations. (a) São Paulo state representation showing locations of the ex situ (1—red circle) and in situ (2 and 3—black circle) populations. (b) Topographic map of the Ilha da Queimada Grande, São Paulo State, Brazil—use authorized by Martins et al. (2008)—with modifications, showing sampling sites (2—yellow circle; 3—light blue circle)

A total of 80 samples from representatives of *B. insularis* (Figure 1) were used in this study: 49 specimens belonging to an ex situ population maintained at the Laboratório de Ecologia e Evolução (LEEV), Instituto Butantan (São Paulo State, Brazil; sampling area 1; Figure 2a; Appendix S1), 31 specimens belonging to the in situ population from Ilha da Queimada Grande (São Paulo State, Brazil; Figure 2b; Appendix S1), 12 of them were taken in sampling area 2, and 19 in sampling area 3 (Figure 2b). We are using sampling area terminology because of the low accuracy of GPS data in the island, so the centroid point from each area was used as the geographic coordinate of the island representatives.

### 2.2 Microsatellites amplification and genotyping

Genomic DNA was extracted from the ventral scales or liver, according to Walsh et al. (1991). Thirty‐three pairs of primers developed for three species of the *B. neuwiedi* group (*B. marmoratus*—Bmar; *B. mattogrossensis*—Bmat; and *B. pauloensis*—Bpau) were used to amplify DNA sequences of *B. insularis* samples (Appendix S2). Loci were amplified with a final volume of 15 µl comprising 3.993 µl milli‐Q water, 1.5 µl 10x PCR buffer, 0.45 µl MgCl_2_ (50 mM), 0.282 µl dNTPs (5 mM), 0.4 µl forward primers with a M13 tail (1 mM), 0.4 µl reverse primers (10 mM), and 0.4 µl of a M13 primer (FAM‐, NED‐, PET‐, or VIC‐ labeled; 10 mM), 0.075 µl Platinum Taq polymerase (1 U/µl; Invitrogen), and 7.5 µl DNA template at a concentration of 60 ng/µl. Reactions were performed on a LifeEco Bioer Thermocycler as described by Machado (2015), but the annealing was reduced to 45 and 30 s in the touchdown and normal cycles, respectively. Microsatellite fragment lengths were obtained through a capillary sequencer (Prism 3730XL Genetic Analyzer; Applied Biosystems) at the “Centro de Pesquisas sobre o Genoma Humano”, Instituto de Biociências, Universidade de São Paulo (IB‐USP). Each microsatellite fragment was scored using the GENEIOUS v.7.1.7 software (Kaerse et al., 2012).

### 2.3 Genetic analyses

Details and the script used for the analysis performed in the software R v.3.5.1 (R Core Team, 2018) are available in the Appendix S3. The following sections will focus on reporting the parameters, packages, and software used. Critical probability values (*p* ≤ .05) for all tests described herein were adjusted with a sequential Bonferroni correction (Rice, 1989).

### 2.4 Data quality control, linkage disequilibrium, and Hardy–Weinberg equilibrium tests

Missing percentages were accessed through POPPR v.2.8.1 package (Kamvar et al., 2014, 2015), and loci and individuals with more than 30% missing data were excluded from the analyses. The occurrence of null alleles and shuttering was investigated using the software Micro‐Checker v.2.2.3 (VanOosterhout et al., 2004). Disequilibrium linkage tests were performed pair‐by‐pair for each population and a matrix containing all data using ADEGENET v.2.1.1 package (Jombart, 2008). Hardy–Weinberg equilibrium (HWE) test was performed in PEGAS v.0.11 package (Paradis, 2010) with 1,000 permutations.

### 2.5 Genetic diversity indexes

The number of alleles (A) and the number of private alleles (PA) were calculated using POPPR. Allelic richness was obtained using HIERFSTAT v.0.44‐22 package (Goudet, 2005). Additionally, the observed and expected heterozygosity indexes were inferred through the basic package implemented in *R*.

### 2.6 Inbreeding indexes

The literature presents two different inbreeding coefficients that possess distinct biological meanings. The inbreeding coefficient (F_is_) evaluates the mating system in the population, and the inbreeding coefficient of the genogram or per kinship (f_is_) assesses the individual probability of exhibiting an allele that is identical by descent (IBD) due to its parental relationships (Templeton, 2011).

Since both biological meanings offer different insights for conservation, we investigated the inbreeding coefficient for the mating system (F_is_) using the FSTAT v.2.9.3.2 software (Goudet, 2002), and the average individual inbreeding coefficient per kinship (f_is_) in each population using ADEGENET. Significance of each index was obtained using bootstrap, with 1,000 permutations.

### 2.7 Bottleneck in the in situ population

The Bottleneck software (Piry et al., 1999) was used to test for a recent demographic reduction in the in situ population. The analysis was performed with 1,000 iterations under two models: the infinite allele model (IAM) and the stepwise mutation model (SMM). Sign up and Wilcoxon tests were used to infer recent bottleneck signals, through significant heterozygosity excess.

### 2.8 Populational genetic structure

Offspring representatives from the ex situ population were excluded of the genetic differentiation and structure analyses in order to avoid bias.

Populational differentiation coefficients are sorted in three classes: (a) the classical Wright's F_st_, (b) the standardized analogues F_st_, and (c) D_est_. Though none of these indexes are considered an ideal summary statistic, combining them may increase accuracy in attempts to elucidate demographic and genetic population structure (Meirmans & Hedrick, 2011). Wright's F_st_ was calculated using FSTAT. Hendrick's G_st_' and Jost's D_est_ indexes were obtained using MMOD (Winter, 2012). AMOVA was performed with POPPR package. Significances of each index and AMOVA were obtained using bootstrap with 1,000 permutations.

Genetic structure within and among populations was analyzed with two approaches: (a) the Bayesian clustering method applied in STRUCTURE v.2.3.4 (Pritchard et al., 2000) and (b) the Discriminant Analysis of Principal Components (DAPC; Jombart et al., 2010) implemented in the ADEGENET v.2.1.1 (Jombart, 2008).

STRUCTURE analyses were conducted without origin prior and testing an admixture model. Initially, we performed ten independent runs for one cluster (*K* = 1) to address the best allele model, and initial *λ* value to be used in the analyses (Janes et al., 2017; Porras‐Hurtado et al., 2013). Then, we investigated the probability of different number of clusters (*K*) from one to six, with a burn‐in of 500,000, followed by 1,000,000 Markov Chain Monte Carlo (MCMC) steps, repeated 30 times. The most probable number of genetic clusters was investigated through the comparisons of the: (a) medium α values obtained for the 30 runs of each *K*; (b) the symmetry distribution of each cluster in the plot generated for each *K*; (c) the Prob (Lnǀ*K*) Method; and (d) the Evanno Method (Evanno et al., 2005) performed in STRUCTURE HARVESTER (Earl, 2012). Data were summarized with Clumpp v.1.1.2 (Jakobsson & Rosenberg, 2007) and visualized with Distruct v.1.1 (Rosenberg, 2004).

For the DAPC analysis, the number of clusters (*K*) was estimated from one to ten using the lowest value of the Bayesian information criterion (BIC) retaining 30 principal components (PCs) using ADEGENET. We increased the numbers of possible *K* to 10 to improve the visualization of the plot obtained for BIC values. Subsequently, we conducted an initial DAPC analysis with parameters of 25 PCs, three discriminant analyses, and assuming *K* number of genetic clusters obtained in the step above. Once bias became possible as a result of the excess of PCs used, the correct number of PCs (i.e., six) was accessed, and the results were validated via the a‐scored method.

### 2.9 Genetic and breeding management of the ex situ *B. insularis* population

Kinship coefficient values were inferred for 37 captive individuals, 34 of which were alive at the final stage of this study, through the ML‐Relate software (Kalinowski et al., 2006). Based on these values, we analyzed (a) the relationship between each pair of possible reproductive individuals, based on Wang (2011), and (b) two cases of uncertain relationship in the captive population. The first case was of uncertain paternity among the possible fathers (ID0001 and ID011F) and three offspring (ID06FF, ID08FF, and ID09FF); the second case was related to the uncertain maternity among the founder females and five offspring (ID011F, ID013F, ID014F, ID015F, and ID021F).

## 3 RESULTS

After data quality, linkage disequilibrium and null alleles tests have been performed, a total of 21 loci and four individuals had to be excluded from the study (Appendix S4) because heterologous amplification was not successful in eleven loci; excessive mistyping and missing data (>30%) were found in three loci and four individuals; evidence of linkage disequilibrium and null alleles were recovered in two and five loci, respectively. Therefore, populational analyses were conducted on the basis of a genetic matrix (Appendix S5) containing 79 individuals (49 ex situ and 30 in situ) and 12 loci (namely Bmar_076, Bpau_002, Bpau_014, Bpau_059, Bpau_083, Bpau_130, Bmat_010, Bmat_049, Bmat_060, Bmat_070, Bmat_080, Bmat_106).

After sequential Bonferroni correction, nine loci (four of the in situ population; five of the ex situ population) indicate a significant deviation from the HWE (*p* ≤ .004), evincing that the populations did not show a congruent pattern in HWE departure. However, the only locus that showed HWE deviation in both the ex situ and in situ populations was Bmat_106; therefore, it was removed from subsequent analyses to avoid bias.

### 3.1 Genetic diversity

The final 11 microsatellites showed similar values of genetic diversity indexes between the populations (Table 1). The number of alleles (A) varied from 2–6 for each locus considering both populations, with a total of 37 and 36 alleles in the ex situ and in situ populations, respectively. Allele richness (AR) ranged from 1.95 to 5 considering both populations. Moreover, the ex situ and in situ populations contain 10 and nine private alleles, respectively. Observed (Ho) and expected heterozygosity (He) were, on average, similar to both populations (Ho*_ex situ_* =0.59, Ho*_in situ_* = 0.53, He*_ex situ_* = 0.48, and He*_in situ_* = 0.47), with the lowest heterozygosity indexes in the locus Bmat_080 in both populations.

**Table 1:** Microsatellite diversity indexes for ex situ and in situ populations of *Bothrops insularis*

Locus	Repeat Motif	Populations											
		Ex situ						In situ					
		A	AR	PA	Ho	He	F_is_	A	AR	PA	Ho	He	F_is_
Bmar_076	(GAG)_n_	2	2.00		0.63	0.44	–0.45	2	2.00		0.60	0.51	−0.19
Bpau_002	(TCTAC)_n_	4	3.40	2	0.82	0.54	–0.54	3	2.98	1	0.78	0.55	−0.44
Bpau_014	(CCAT)_n_	2	2.00		0.56	0.45	–0.26	4	3.92	2	0.40	0.45	0.12
Bpau_059	(ATCC)_n_	6	5.00	2	0.75	0.70	–0.07	4	4.00		0.74	0.71	0.05
Bpau_083	(TCA)_n_	4	3.55	1	0.61	0.46	–0.32	3	2.96		0.42	0.39	−0.06
Bpau_130	(GAG)_n_	2	2.00		0.47	0.47	0.02	2	2.00		0.40	0.33	−0.23
Bmat_010	(ATGG)_n_	5	4.48	1	0.95	0.64	–0.49	4	3.96		0.55	0.52	−0.06
Bmat_049	(TGGA)_n_	3	2.51	1	0.62	0.44	–0.42	2	2.00		0.23	0.35	0.33
Bmat_060	(CCAT)_n_	5	4.81	2	0.68	0.73	0.07	5	5.00	2	0.67	0.71	0.08
Bmat_070	(TCCA)_n_	2	2.00		0.32	0.30	–0.06	4	3.76	2	1	0.57	−0.78
Bmat_080	(GAG)_n_	2	1.95	1	0.09	0.09	–0.04	3	2.76	2	0.03	0.13	0.74
Average		3.36	3.06		0.59	0.48	–0.24	3.27	3.21		0.53	0.47	−0.12

Note

A = Number of alleles; AR = Allele richness; PA = Number of private alleles; Ho = Observed heterozygosity; He = Expected heterozygosity; F_is_ = Inbreeding mating system coefficient.

### 3.2 Inbreeding

We obtained negative and significant average values of F_is_ for both populations (F_is ex situ_ = −0.24/CI = −0.43 < *x* < −0.06 and F_is in situ_ = −0.12/CI = −0.43 < *x *< 0.09; Table 1). However, both populations showed positive f_is_ values, which were only significant in the in situ population (f_is ex situ _= 0.05/CI = –0.42 < *x* < −0.02 and f_is in situ_ = 0.06/CI = −0.40 < *x* < 0.10).

### 3.3 Bottleneck

Under the models tested, the results obtained showed no evidence of a recent bottleneck in the in situ population after Bonferroni sequential correction (IAM: 0.03; SMM: 0.55). However, it is worth mentioning that under the IAM model, nine out of 11 loci showed heterozygosity excess.

### 3.4 Populational differentiation indexes

Levels of genetic differentiation between the populations are presented in Table 2. All of the genetic differentiation indexes recovered showed, on average, low but nevertheless significant values (F_st_ = 0.07/CI = 0.01 < *x* < 0.13, G_st_^' ^= 0.12/CI = 0.05 < *x* < 0.20, D_est_ = 0.06/CI = 0.02 < *x* < 0.10), which was corroborated by AMOVA results (variation between populations = 12%; *p* < .001). However, some loci show moderate to high values of genetic differentiation indexes (for instance, Bmat_070)—it seems to be related to both the existence of private alleles (Table 1) and the difference between the allelic frequencies within the populations (Appendix S5).

**Table 2:** Genetic differentiation indexes of the ex situ and in situ *Bothrops insularis* populations

Locus	F_st_	G_st_'	D_est_
Bmar_076	0.098	0.179	0.092
Bpau_002	–0.007	0.000	0.000
Bpau_014	–0.005	0.000	0.000
Bpau_059	–0.010	0.000	0.000
Bpau_083	0.035	0.047	0.023
Bpau_130	0.035	0.042	0.017
Bmat_010	0.089	0.191	0.124
Bmat_049	–0.026	0.000	0.000
Bmat_060	0.077	0.293	0.230
Bmat_070	0.316	0.543	0.297
Bmat_080	0.015	0.026	0.004
Average	0.07 (0.01 < *x* < 0.13)	0.12 (0.05 < *x* < 0.20)	0.06 (0.02 < *x* < 0.10)

Note

F_st_ = Wright's genetic differentiation index. G_st_ = Hendrick's F_st_ analogous. D_est_ = Jost's genetic differentiation index.

Values between parentheses indicate the confidence interval obtained through 1,000 replications with bootstrap method.

### 3.5 Genetic structure

**Figure 3:**
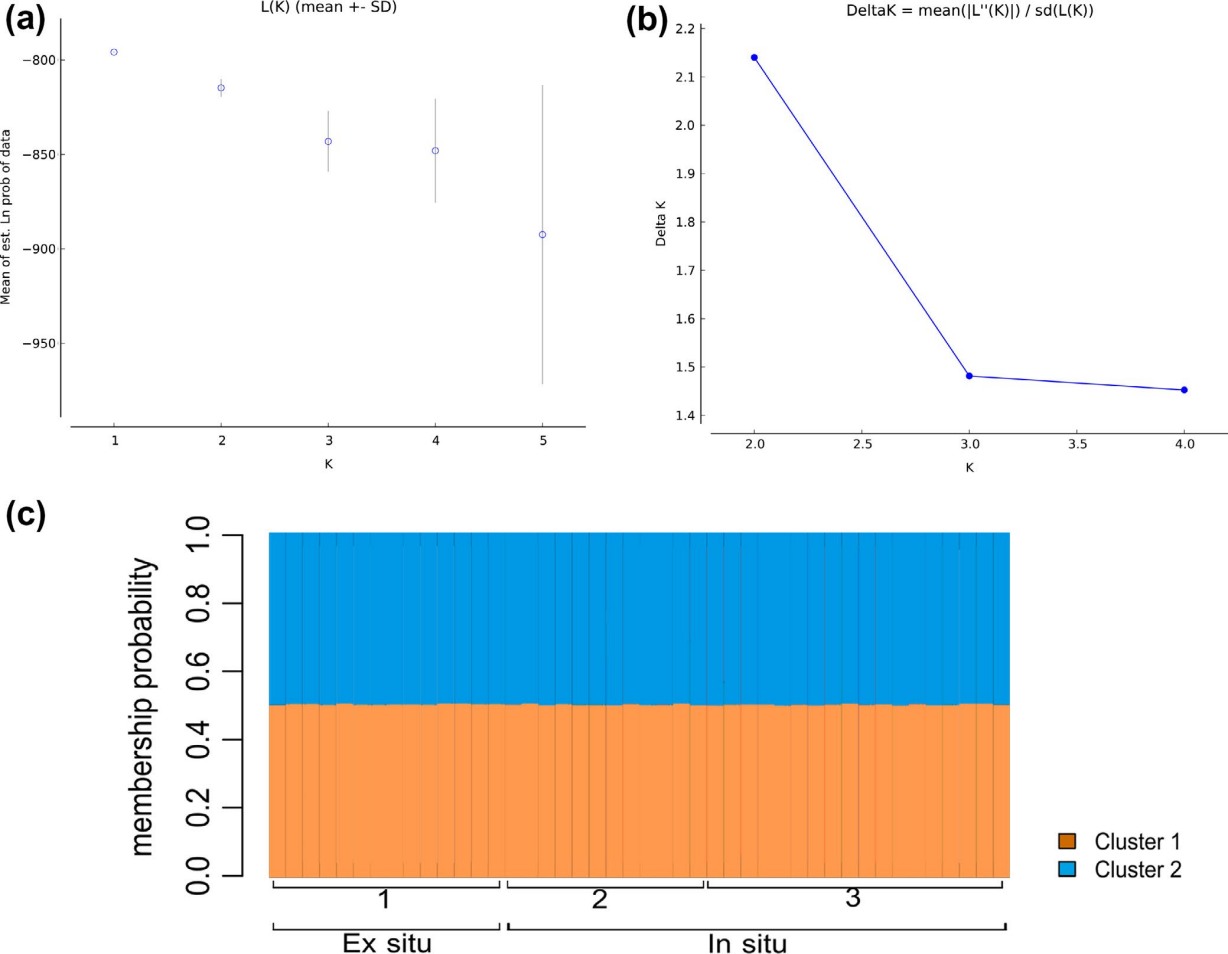
Genetic structure of *Bothrops insularis* using 11 microsatellite loci and the STRUCTURE software. (a) Graphic of the likelihood as a function of *K* (Prob (Lnǀ*K*) Method; best: *K* = 1). (b) Graphic of the ΔK for *B. insularis* (Evanno method; best *K* = 2). (c) Bar plot showing the membership probability of each individual to belong to each genetic cluster (*K* = 2).

STRUCTURE analysis suggests the existence of one or two clusters according to the method used. The Prob (Lnǀ*K*) Method recovered one genetic cluster (*K* = 1; Figure 3a). On the other hand, based on the Evanno Method, we recovered two clusters (Δ*K* = 2; Figure 3b). Janes et al. (2017) highlight an inconsistency on the Evanno Method arguing that it has a tendency of choosing *K* = 2 as the most probable number of clusters, even when it is not true. Therefore, the authors and the STRUCTURE manual suggest that other parameters, such as the *α* value and the clusters distribution on the plots, should be revised to address the correct number of clusters considering the dataset. For all *K* tested, we recovered *α* > 1 and a highly symmetric clusters distribution on the plots generated, which indicates no existence of subpopulations (Figure 3c).

**Figure 4:**
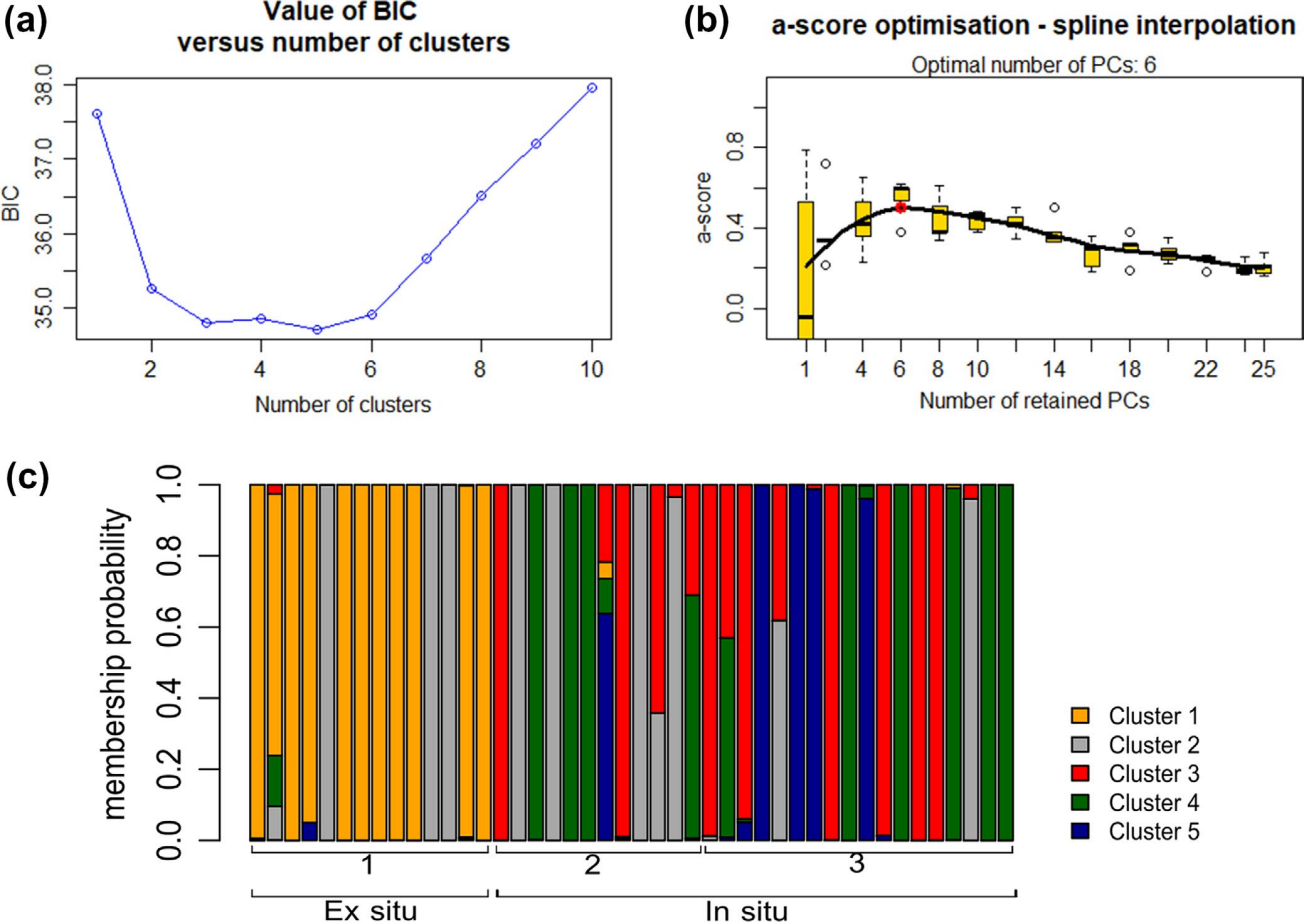
Genetic structure of *Bothrops insularis* using 11 microsatellite loci and the Discriminant Analysis of Principal Components. (a) Graphic of the Bayesian Information Criterion (BIC) as a function of *K* (best: *K* = 5). (b) Graphic of the α‐scored as a function of number of Principal Components (PC; best: 6 PCs). (c) Bar plot showing the membership probability of each individual to belong to each genetic cluster (*K* = 5). Note the existence of one cluster exclusive to the ex situ population (yellow) and the absence of correlation of the sampling location and clusters obtained in the in situ population (red, green, blue, and gray).

DAPC analysis indicates the existence of five clusters according to BIC values (*K* = 5, Figure 4a), suggesting that there are genetic clusters no recovered in STRUCTURE. Thereafter, the DAPC recovered four clusters that are exclusive to one population, represented by yellow for the ex situ population and by red, green, and blue for the in situ population (Figure 4c). None spatial relationships among the clusters in the island population were found (Figure 4c).

### 3.6 Genetic and breeding management of the ex situ *B. insularis* population

We recovered a mean kinship value of 0.20 for the captive population based on the weighted average of the mean relatedness values from the 34 alive individuals (Appendix S6). Furthermore, the genetic data allowed the two cases of uncertain paternity and uncertain maternity to be resolved. Due the decease of the putative father ID0001 and absence of any tissue for accessing any genetic information, we were not able to test his paternity. Then, paternity analyses were only conduct using the possible father ID011F, revealing relatedness values (*R*) equal to zero among this possible father and the three offspring (ID06FF, ID08FF, and ID09FF; Table 3; Appendix S7), excluding this possible parentage. Maternity analyses revealed a founder female as mother for each offspring (Table 3; Appendix S7): mother ID0009 to the offspring ID011F, mother ID0010 to the offspring ID013F, mother ID0010 to the offspring ID014F, mother ID0006 to the offspring ID015F, and mother ID0013 to the offspring ID021F. Individuals ID06FF, ID013F, and ID021F were excluded from Appendix S6, given that they are dead, therefore unable to contribute as relatives to the next generations. After addressing these uncertain parental relationships, a pedigree of the ex situ population was drawn, assembling these data with the previous breeding information (Appendix S7).

**Table 3:** Parentage analyses of offspring with uncertain paternity and unknown mother; *p*–values obtained through the ML Related analyses

Putative Parent	Offspring							
	ID06FF	ID08FF	ID09FF	ID011F	ID013F	ID014F	ID015F	ID021F
ID011F	1.000	0.170	0.687	–	–	–	–	–
ID0003	–	–	–	0.169	0.108	0.123	0.065	0.073
ID0005	–	–	–	0.186	0.318	0.629	0.192	0.188
ID0006	–	–	–	1.000	0.043	0.110	0.054^a^	0.125
ID0007	–	–	–	0.190	1.000	0.138	1.000	1.000
ID0008	–	–	–	0.195	1.000	1.000	1.000	0.200
ID0009	–	–	–	0.146^a^	0.966	0.177	0.639	0.179
ID0010	–	–	–	0.728	0.026^a^	0.024^a^	0.147	0.173
ID0011	–	–	–	0.653	1.000	0.968	0.810	0.814
ID0012	–	–	–	0.900	1.000	1.000	0.104	0.049
ID0013	–	–	–	0.881	1.000	1.000	0.097	0.046^a^

^a^ Representatives with the major probability of maternal relationship with offspring.

Aiming to improve the genetic contribution of each founder, and to avoid inbreeding, six preferential couples are suggested (Table 4).

**Table 4:** List of suggested breeding couples based on microsatellite data

Male	Mean kinship	Female	Mean kinship	*R*
ID0018	0.25	ID0007	0.17	.06
ID0017	0.15	ID014F	0.11	0
ID009F	0.12	ID001F	0.18	0
ID27FF	0.18	ID09FF	0.14	.06
ID11FF	0.12	ID03FF	0.19	0
ID19FF	0.17	ID08FF	0.13	0

Note

*R* = Relatedness index value between each pair of representatives.

## 4 DISCUSSION

Conservation studies and efforts have been predominantly focused on endangered species (McLennan et al., 2018; Reynolds et al., 2015; Rodriguez et al., 2012). Accordingly, this study is a contribution to the conservation program of *B. insularis*, a critically endangered Brazilian snake, for which we describe the first genetic diversity framework of a wild and a captive population. Genetic parameters presented herein are required by the National Conservation Plan of Insular Herpetofauna (Bataus et al., 2011) and should guide the delimitation of conservation strategies. Besides the fact that the species is included in Brazilian conservation plans, *B. insularis* should be conserved because of: (a) its diversification process and phylogenetic relationships within the *B. jararaca* group (Alencar et al., 2016; Grazziotin et al., 2006; Hamdan et al., 2020); (b) the uniqueness of ecological and reproductive traits, such as its diet based on birds as adults and the hemipenis in females (Hoge et al., 1953; Marques et al., 2012; Martins et al., 2002), its unique venom which toxicity changes ontogenetically and is higher upon birds than mammals (Zelanis et al., 2008), and the possibility of using this species as a model for evolutionary and ecological studies (Duarte et al., 1995).

### 4.1 Population genetics of *B. insularis*

Heterologous amplification is a tool widely used in studies of conservation genetics. The success of heterologous amplifications is higher in closely related species relative to distant species (Blouin‐Dermers & Gibbs, 2003; Mattson et al., 2007; Simonov & Wink, 2011). A high rate of heterologous amplification was expected in this study given that *B. insularis* is included in the *B. jararaca* complex, which is the sister group to the *B. neuwiedi* complex (Carrasco et al., 2012). We recorded a success rate of 59.3% heterologous amplification, in which *B. mattogrossensis* microsatellite loci performed better than *B. pauloensis* and *B. marmoratus* loci. This evinces the efficacy of such markers when studying populations within the *B. jararaca* complex, despite not being specific to this group.

Isolated, small, and critically endangered populations tend to present low values of genetic diversity and inbreeding occurrence relative to continental or unthreatened populations (Allendorf et al., 2013; Frankham et al., 2008). Considering the number of alleles and the heterozygosity rates, genetic diversity in each population was low compared with *B. neuwiedi* group species (*B. marmoratus*, *B. mattogrossensis*, and *B. pauloensis*; Machado, 2015) and other continental species (Duan et al., 2017; King, 2009). Low genetic diversity was also recovered in other critically endangered snakes (Jaeger et al., 2016; King, 2009; Meister et al., 2012; Wang et al., 2015); heterozygosity values found herein were similar to the ones compiled by King (2009) for snake species (0.35–0.87/ average: 0.60). The exception is the locus Bmat_080, which show the lowest rates of heterozygosity (ex situ: 0.09*;* in situ: 0.03–0.13), possibly as a consequence of a fixation process of the allele “193” (Appendix S5).

The higher rates of observed heterozygosity in comparison with the expected heterozygosity might be explained by the association of demographic events and the genetic diversity. Founder effects and bottlenecks are known to the phenomena responsible for the reduction of the genetic diversity of populations, leading to a reduction in the number of alleles and heterozygosity (Piry et al., 1999). However, reduction in the number of alleles is faster than the decrease in heterozygosity rates, so that in some cases observed heterozygosity rates may be higher than the expected ones, which are based on the allele frequencies (King, 2009; Luikart & Cornuet, 1998). It might be considered that populational growth after a bottleneck event may increase heterozygosity rates (Allendorf et al., 2013). Demographic history records of *B. insularis* indicate that the in situ population declined in the past 20 years due to bushfires and anthropogenic actions. The last populational census recovered a high density and stability in the in situ population, with records of growth rates (Guimarães et al., 2014). Therefore, even though our results did not recover evidences of a recent bottleneck, we may hypothesize that the heterozygosity rates obtained herein might be explained by the existence of bottleneck effect during the last two decades (or the founder effect in the ex situ population) associated with demographic growth in both populations during the last years.

We recovered a significant negative value of inbreeding mating system coefficient for both ex situ and in situ populations. These results contradict the tendency for positive inbreeding values observed in snakes (see King, 2009). Though the analyses indicate that outbreeding is the mating system in both populations, the in situ population showed a significant positive value of kinship inbreeding coefficient, suggesting the occurrence of endogamy, which is the mating of related individuals (Allendorf et al., 2013). Endogamy was also reported in another endemic insular snake, *Gloydius shedaoensis* (Wang et al., 2015). In their study, the authors discuss the possibility that this species evolved dispersal strategies to avoid endogamy. Likewise, we hypothesize that the outbreeding mating system could also have evolved in *B. insularis* as a response to endogamy.

Furthermore, the existence of inbreeding may reduce the genetic fitness in the population, leading, in some cases, to a decline in fecundity and survival rates, as well as sexual abnormalities. Reproductive studies in *B. insularis* have been reported hemipenis in different developmental stages in females (Hoge et al., 1953; Kasperoviczus, 2009), small fecundity of this species when compared to its mainland relative *B. jararaca* (Marques et al., 2013), and high level of mutations in males sperm which lead to a reduction in the number of the viable ones (Silva et al., 2015). Therefore, even though we used neutral molecular markers, the data generated herein may be considered an initial step to associate molecular and reproductive data on *B. insularis*.

The genetic divergence between populations could be explained by the existence of private alleles, which, associated with the founder effect of the ex situ population and random sampling of the in situ population, could lead to the low but significant genetic differentiation observed. STRUCTURE results suggest the existence of one population, while DAPC recovered five distinctive genetic clusters with no geographical correlation to the sampling areas. A recent study on the Cascade red fox (*Vulpes vulpes*) also revealed the existence of clusters with no geographic relationship, suggesting family lineages within representatives as a possible explanation. The authors proposed that the mixture of these clusters in the space could indicate the existence of gene flow (Akins et al., 2018). Furthermore, analyses performed in *G. shedaoensis* showed the existence of gene flow among subpopulations, with relatives found over a wide geographical spread, allowing these subpopulations to be considered a single conservation unit (Wang et al., 2015). Based on this, although the studies concerned different species with different vagilities, we could hypothesize that there is no structure within the *B. insularis* populations, and the genetic clusters found in DAPC should be related to (a) the isolation of the ex situ population, and (b) existence of recognized families within the wild population due to the low vagility observed (personal observation, Karina Banci). Besides, the gene flow detected based on the pattern distribution of the family lineages suggests that the island might also be considered a single conservation unit.

### 4.2 Conservation implications

Although most of the ex situ conservation studies focused only on captive populations, recent researches have highlighted the importance of monitoring in situ populations prior to assessing captive populations, and the integration of both procedures to improve conservation strategies (Castellanos‐Morales et al., 2016; Frankham, 2010; Witzenberger & Hochkirch, 2011). Additionally, the so‐called sampling address of the founder individuals must be selected in order to minimize inbreeding and outbreeding effects in the ex situ population (Újvari et al., 2002), taking into account that it should retain 90%–95% genetic similarity to its wild counterpart (Castellanos‐Morales et al., 2016; Frankham et al., 2008). We observed a mere 49% of genetic similarity between the in situ and ex situ populations, explained by the low but significative divergence between them due to distinct allelic frequencies. Therefore, we strongly suggest that new individuals from the island should be incorporated into the ex situ population; besides, a breeding protocol (based on genetic and reproductive information) should also be established, in order to choose the ideal couples which may reduce such biases, maintaining the genetic similarity between the populations, as well as avoiding loss of genetic diversity and inbreeding. We also recommend that these data should be associated with ecology and reproductive information guiding future research on this species, so that the genetic data will be used as a base to maintain a healthy and viable captive population, with highly genetically similar to the in situ one, which is crucial for future reintroductions.

Several reproduction and management strategies are proposed to maintain genetic variability and to avoid inbreeding and captive adaptations in the ex situ population. Fernández and Caballero (2001) pointed out that strategies focusing on the reduction of mean kinship among individuals, dampening of the founder effect, or a combination of both strategies are widely used in ex situ conservation programs. Another important issue that must be accounted for is the lack of parentage information for some individuals, which may bias management strategies due to underestimation of kinship and inbreeding within populations (Jiménez‐Mena et al., 2015). Our results allowed us to refute a potential relationship between a putative father and three descendants and identify four maternal founders related to five descendants, whose mothers were undetermined. The average kinship value suggests that the ex situ population is composed of half‐siblings and representatives with small parentage degree levels (e.g., cousins, uncles, nephews, grandparents), which can be explained by the high genetic contribution of few founders (see Appendix S6). Based on the kinship values and the strategies cited above, we suggest six preferential crosses to the ex situ population (Table 4).

Importantly, a significant inbreeding coefficient was found in the in situ population. Studies have shown that inbreeding is correlated with the fixation of deleterious genes and, when reaching inbreeding depression, results in extinction (Allendorf et al., 2013; Brook et al., 2002; Frankham et al., 2008). Therefore, continuous monitoring of genetic parameters and delimitation of strategies to reduce inbreeding is critical to guarantee the successful conservation of the in situ population, as well as to access genes which may be related to the fitness of this species.

Due to the importance of this species, the Laboratório de Ecologia e Evolução of the Instituto Butantan is developing an interdisciplinary conservation program aiming to implement successful conservation strategies for this species. So far, researchers’ focus was mainly reproduction (sperm viability — Silva et al., 2015; mating season — Marques et al., 2013; Amorim et al., 2019; correlation between diet, development, and sexual maturity — Passos, 2018;) and ecology (diet — Marques et al., 2012; natural history — Marques et al., 2002; populational census — Martins et al., 2008; Guimarães et al., 2014). Our study marked the introduction of genetic studies to guide the management of this species, demonstrating the importance of these parameters to trace conservation strategies, and providing a basis for comparative studies in the future, as well as for the development of conservation efforts. Furthermore, in order to look the hypotheses discussed herein, an integrative study focusing on the home range, dispersal capacity, and correlation of kinship and geographic distance of representatives has being carried out in our laboratory.

It is also worth mentioning that all the information generated herein should provide the base of the development and implementation of the conservation center of the Instituto Butantan’ Zoo Park.

## 5 CONCLUSIONS

*Bothrops insularis* showed levels of genetic diversity similar to those expected for insular, small, and isolated populations. Genetic differentiation between both in situ and ex situ populations could be related to genetic drift, founder effect, and sampling gaps in the ex situ population foundation. Though the inbreeding coefficient was only significant in the in situ population, the screen of this parameter is crucial to any conservation strategy for this species. Overall, to improve the management of the in situ and ex situ populations, we suggest the following: (a) screening the loss of genetic diversity in the ex situ populations; (b) incorporating new representatives from in situ population as a method to improve genetic diversity and maintain genetic homogeneity between populations; (c) following preferential breeding pairs to avoid inbreeding and to guarantee equal genetic contribution of all founders; and (d) analyze possible impacts of outbreeding if translocations and reintroductions are stablished as conservation purposes of the ex situ population. In sum, this study is a preliminary contribution to the conservation program of *B. insularis* and might be used as initial mark for comparative in future genetic assessments, as well as in studies with ecology and reproduction for this and related species.

## CONFLICT OF INTEREST

The authors have knowledge about the content within this manuscript and declare the absence of conflict interest.

## AUTHOR CONTRIBUTION

**Igor Salles‐Oliveira:** Conceptualization (equal); Data curation (lead); Formal analysis (lead); Investigation (equal); Methodology (lead); Validation (lead); Visualization (equal); Writing‐original draft (lead); Writing‐review & editing (lead). **Taís Machado:** Data curation (supporting); Formal analysis (supporting); Methodology (supporting); Writing‐original draft (supporting); Writing‐review & editing (supporting). **Karina Banci:** Resources (supporting); Writing‐original draft (supporting); Writing‐review & editing (supporting). **Selma Maria Almeida‐Santos:** Resources (supporting); Writing‐review & editing (supporting). **Maria José de J. Silva:** Conceptualization (lead); Data curation (equal); Formal analysis (equal); Funding acquisition (lead); Investigation (supporting); Methodology (supporting); Project administration (lead); Resources (lead); Supervision (lead); Validation (supporting); Visualization (equal); Writing‐original draft (supporting); Writing‐review & editing (supporting).

## ETHICAL APPROVAL

The completion of this project and sample collection were performed under license and authorization of the Ethics Committee on the Use of Animals from Instituto Butantan (CEUAIB – no. 9813/12, CEUAIB – no. 3124270671/17, and CEUAIB – no. 15431705/18).

## Supporting information

AppendixS1

AppendixS2

AppendixS3

AppendixS4

AppendixS5

AppendixS6

AppendixS7

## Data Availability

All data underlying this manuscript are available online on Dryad (doi.org/10.5061/dryad.bvq83bk6r). Dataset description: (a) Geographic information of the samplings: Appendix S1, (b) list of the 33 microsatellites tested: Appendix S2, (c) *R*‐script used in the analysis: Appendix S3; (d) Simplified scheme of the methodology: Appendix S4; (e) genotype data obtained from PCR amplification of the 12 loci for the 80 representative samples used herein: Appendix S5; (e) table of the kinship value obtained for each pair of individuals that was alive (*n* = 34): Appendix S6, (f) pedigree from the ex situ population of *B. insularis* housed at the Laboratório de Ecologia e Evolução, Instituto Butantan, São Paulo state, Brazil: Appendix S7.
