# Supplementary material for: Genetic variability, management, and conservation implications of the critically endangered Brazilian pitviper Bothrops insularis
Source: Ecol Evol. 2020 Oct 3;10(23):12870–82. doi: 10.1002/ece3.6838 (PMC7713924; doi:10.1002/ece3.6838)
Supplement: Supplementary file 1 — AppendixS1 [file ECE3-10-12870-s001.docx]

# Appendix S1

**Article title:** Genetic variability, management, and conservation implications of the critically endangered Brazilian pitviper *Bothrops insularis*

**Journal name:** Ecology and Evolution

**Author names:** Igor Salles de Oliveira, Taís Machado, Karina Banci, Selma Maria Almeida-Santos, and Maria José de J. Silva.

**Corresponding author:** Maria José de J. Silva.

**Affiliation:** Laboratório de Ecologia e Evolução – Instituto Butantan, Av. Dr. Vital Brazil, 1500 – 05503-000 – São Paulo, SP, Brazil.

**E-mail:** mariajose.silva@butantan.gov.br

# List of representatives from the *Bothrops insularis* *ex-situ* (captive) population housed in the Laboratório de Ecologia e Evolução, Instituto Butantan, São Paulo State, Brazil. The suffixes F and FF refer to individuals born in 2010 and 2013/2014, respectively.

# Sampling area 1 (Fig. 2A), geographical coordinates: –23.3351; –46.4315.

**Males**: ID0002, ID0004, ID0016, ID0017, ID0018, ID002F, ID004F, ID005F, ID006F, ID0009F, ID011F, ID013F, ID021F, ID024F, ID01FF, ID11FF, ID12FF, ID13FF, ID14FF, ID19FF, ID20FF, ID23FF, ID26FF, ID27FF.

**Females**: ID0003, ID0005, ID0006, ID0007, ID0008, ID0009, ID0010, ID0011, ID0012, ID0013, ID001F, ID014F, ID015F, ID017F, ID022F, ID02FF, ID03FF, ID08FF, ID09FF, ID16FF, ID22FF.

**Unknown Sex**: ID04FF, ID05FF, ID06FF, ID21FF.

**Online resource 2**

List of representatives from the *Bothrops insularis* *in-situ* (Ilha da Queimada Grande) population, São Paulo State, Brazil.

Sampling site 2 (Fig. 2A, B), geographical coordinate of the centroid point: **–**24.47996111; **–**46.67593611.

**Males**: KB0001, KB0011, KB0012, KB0020, KB0021, KB0029, KB0030.

**Females**: KB0002, KB0003, KB0013, KB0014, KB0028.

Sampling site 3 (Fig. 2A, B), geographical coordinate of the centroid point: **–**24.48476944; **–**46.67585000.

**Males**: KB0007, KB0018, KB0019, KB0022, KB0024, KB0026, KB0031.

**Females**: KB0004, KB0005, KB0006, KB0008, KB0009, KB0010, KB0015, KB0016, KB0017, KB0023, KB0025, KB0027.
