# Supplementary material for: Genetic variability, management, and conservation implications of the critically endangered Brazilian pitviper Bothrops insularis
Source: Ecol Evol. 2020 Oct 3;10(23):12870–82. doi: 10.1002/ece3.6838 (PMC7713924; doi:10.1002/ece3.6838)
Supplement: Supplementary file 2 — AppendixS2 [file ECE3-10-12870-s002.docx]

# Appendix S2

**Article title:** Genetic variability, management, and conservation implications of the critically endangered Brazilian pitviper *Bothrops insularis*

**Journal name:** Ecology and Evolution

**Author names:** Igor Salles de Oliveira, Taís Machado, Karina Banci, Selma Maria Almeida-Santos, and Maria José de J. Silva.

**Corresponding author:** Maria José de J. Silva.

**Affiliation:** Laboratório de Ecologia e Evolução – Instituto Butantan, Av. Dr. Vital Brazil, 1500 – 05503-000 – São Paulo, SP, Brazil.

**E-mail:** mariajose.silva@butantan.gov.br

**Table S1** – List of the 33 microsatellite loci (Machado, 2015) tested in this study. The acronyms Bmar, Bmat, and Bpau refer to the primers obtained from *B. marmoratus*, *B. mattogrossensis*, and *B. pauloensis*, respectively.

| **Locus** | **Forward primer** | **Reverse primer** | **Fluorophore** |
| --- | --- | --- | --- |
| **Bmar_005** | TGTAAAACGACGGCCAGTGGCAATTCTTAGCCTGCTTG | GTTTAGCCGTTTGTAATGTCCAGC | FAM |
| **Bmar _019** | TAATACGACTCACTATAGGGCCACCTCAGCTTTTGTCCAT | GTTTAAACGGGAAGGAAGCAAAGT | VIC |
| **Bmar _027** | TGTAAAACGACGGCCAGTGAATGTCTTGCCAAATGGGT | GTTTATTGTTGTTGTGAGCCGTCC | FAM |
| **Bmar _030** | TAATACGACTCACTATAGGGTGGTTCTAAAGCCTGAGCCA | GTTTAGGCGATGTTGCCAACTAAG | VIC |
| **Bmar _065** | GATAACAATTTCACACAGGTTTCTATTTCTTTATCTGTCTG | GTTTCCCTGTGTTAAGATAGGCAGC | PET |
| **Bmar _076** | TTTCCCAGTCACGACGTTGCAAAATGGTCCACTGAGGGT | GTTTGCTATCAAGCAAGGTCTCCC | NED |
| **Bmar _078** | GATAACAATTTCACACAGGCATCTGGTGCGAGCTGTTAG | GTTTGTGTGGTTGACATTGGAGGA | PET |
| **Bmar _079** | TGTAAAACGACGGCCAGTAAATCTGGCTGGCAGAAGAG | GTTTGCAACACTGGGCATCTAAT | FAM |
| **Bmar _087** | GATAACAATTTCACACAGGGGGCAGAATTCCCACAAGTA | GTTTAGAAACAAACCAGCCAACTG | PET |
| **Bmar _102** | TGTAAAACGACGGCCAGTCTCTTTTGCAGTTATGGCCC | GTTTGGCTTAGGAAGACACTGAAA | FAM |
| **Bmar _118** | TTTCCCAGTCACGACGTTGTGGATCTGCAAGCTTCATTG | GTTTCCCTTCCCTTCTCCATTCT | NED |
| **Bmat _009** | TGTAAAACGACGGCCAGTTGTGGTAAGATGAGCAGCCA | GTTTATTCCCGAGTTTCAGTGGTG | FAM |
| **Bmat _010** | TAATACGACTCACTATAGGGTCAGAAGGGGAGAAAGGGAT | GTTTACACAAAAGAGGCCTAGCCA | VIC |
| **Bmat_017** | TTTCCCAGTCACGACGTTGGAAAAGACACATAACTTCAGCCA | GTTTCTGTTGAAAGGGAAGGCAAC | NED |

**Sequel Table S1** – List of the 33 microsatellite loci (Machado, 2015) tested in this study. The acronyms Bmar, Bmat, and Bpau refer to the primers obtained from *B. marmoratus*, *B. mattogrossensis*, and *B. pauloensis*, respectively.

| **Locus** | | **Forward primer** | | **Reverse primer** | **Fluorophore** |
| --- | --- | --- | --- | --- | --- |
| **Bmat _018** | | GATAACAATTTCACACAGGAGGCAAGCATCTTCAGCACT | | GTTTCCTTTCCTTGTACCAAAAGTGA | PET |
| **Bmat _049** | | TGTAAAACGACGGCCAGTAGCCAAGCTCACAGATCAAAG | | GTTTCATTCCTTTGCCACACTCCT | FAM |
| **Bmat _060** | | TAATACGACTCACTATAGGGAAGAATTGACAACTTGATAGC | | GTTTAGGGAGACTGAAGCCAATGA | VIC |
| **Bmat _070** | | TTTCCCAGTCACGACGTTGTGCCTACCGTGACCCTAAAC | | GTTTCCCCTGTCTCTGTCTGTGTT | NED |
| **Bmat _080** | | TGTAAAACGACGGCCAGTTCAGTGAAAATGAGTTGGGTG | | GTTTGGGCATTGTGACTCAAACCT | FAM |
| **Bmat_081** | TAATACGACTCACTATAGGGGCTTTCTGCTCTGCTGGTCT | | GTTTGCTGACCTTCTCTTCTCCCA | | VIC |
| **Bmat _091** | TTTCCCAGTCACGACGTTGCCACACTGCTTGACTGAGGA | | GTTTGCCCAGGAGGTTAAGGAAAC | | NED |
| **Bmat _106** | TTTCCCAGTCACGACGTTGGGATCCACTTCATTCGCCTA | | GTTTGGGCTTGCTTGTTCTTCTTG | | NED |
| **Bpau_002** | TAATACGACTCACTATAGGGGGGAATGTTGGGAACAAGTG | | GTTTAGGGAATTTGTCTCCAGTGC | | VIC |
| **Bpau_014** | GATAACAATTTCACACAGGCATTCCTTTGCCACACTCCT | | GTTTACTTGTCAGCCATGCAAGC | | PET |
| **Bpau_017** | TTTCCCAGTCACGACGTTGAAAGCACATGGCCTCTTCAC | | GTTTGAGGTTAAGATTGTGGGGCA | | NED |
| **Bpau_059** | TTTCCCAGTCACGACGTTGAGCTCAGACGGTACCAAGGA | | GTTTCGCTACAGTTGACAGGCAGA | | NED |
| **Bpau_074** | TTTCCCAGTCACGACGTTGTATCATGGGATTGTCTGGGC | | GTTTGATCTGGCCTGGGTTTAAG | | NED |
| **Bpau_079** | TGTAAAACGACGGCCAGTCACAATCTATTTCAGTCCTGCTG | | GTTTAATCAGTGGAACAACCTGCC | | FAM |
| **Bpau_082** | TTTCCCAGTCACGACGTTGCGGAGACCAAAGGTGGTTAC | | GTTTGGTGGAAGGAGCCTATG | | NED |
| **Bpau_083** | TAATACGACTCACTATAGGGCAAGGTTGTCTTGGTGCCTT | | GTTTCACCAGTTTCAGACTTGGAAGA | | VIC |
| **Bpau_112** | TGTAAAACGACGGCCAGTCCACTGCCCGAATTCTCTAT | | GTTTCTCCTCCCAAACCTTCCT | | FAM |
| **Bpau_130** | GATAACAATTTCACACAGGGTAAAATCAGGGTCAGCCCA | | GTTTGGAAAGGTAACTTCTGCCCA | | PET |
| **Bpau_142** | TAATACGACTCACTATAGGGTCTATTGCTGGTCTATGACCGT | | GTTTCTGCACCACCATAGCTCTCA | | VIC |
