# Supplementary material for: Genetic variability, management, and conservation implications of the critically endangered Brazilian pitviper Bothrops insularis
Source: Ecol Evol. 2020 Oct 3;10(23):12870–82. doi: 10.1002/ece3.6838 (PMC7713924; doi:10.1002/ece3.6838)
Supplement: Supplementary file 3 — AppendixS3 [file ECE3-10-12870-s003.docx]

Appendix S**3**

**Article title:** Genetic variability, management, and conservation implications of the critically endangered Brazilian pitviper *Bothrops insularis*.

**Journal name:** Ecology and Evolution.

**Authors name:** Igor Salles de Oliveira, Taís Machado, Karina Banci, Selma Maria Almeida-Santos, and Maria José de J. Silva.

**Corresponding author:** Maria José de J. Silva

**Affiliation:** Laboratório de Ecologia e Evolução – Instituto Butantan, Av. Dr. Vital Brazil, 1500 – 05503-000 – São Paulo, SP, Brazil.

**E-mail**: [mariajose.silva@butantan.gov.br](mailto:mariajose.silva@butantan.gov.br)

This document presents the script used to perform all analyses done for the article ***Genetic variability, management, and conservation implications of the critically endangered Brazilian pitviper Bothrops insularis*** through the software R v.3.5.1 (RCoreTeam, 2018), using the following packages: POPPR v.2.8.1 (Kamvar, Tabima, & Gruwald, 2014; Kamvar, Brooks, & Grunwald, 2015); ADEGENET v.2.1.1 (Jombart, 2008); PEGAS v.0.11 (PARADIS, 2010); HIERFSTAT v.0.44-22 (Goudet, 2005); MMOD (Winter, 2012); and GGPLOT2 (Wickham, 2016).

#STEPONE – INSTALLING AND ACCESSING PACKAGES.

#Installing packages

install.packages ("poppr")

install.packages ("adegenet")

install.packages ("pegas")

install.packages ("hierfstat")

install.packages ("ggplot2")

install.packages ("mmod")

#Accessing packages

library (poppr)

library (adegenet)

library (pegas)

library (hierfstat)

library (ggplot2)

library (mmod)

#STEPTWO – ACESSING THE GENETIC DATA TO OBTAIN A GENING OBJECT.

Binsularis <- read.genepop ("B_insularis_1pop.gen", ncode = 3, quiet = FALSE)

#STEP THREE – DATA QUALITY, LINKAGE DISEQUILIBRIUM AND HARDY-WEIBERG EQUILIBRIUM TESTS.

## Data quality test

Bins_clean <- poppr::missingno (Binsularis, type = "genotype", cutoff = 0.3)

Bins_clean2 <- poppr::missingno (Bins_clean, type = "loci", cutoff = 0.3)

B_insularis <- Bins_clean2

set.seed(999)

pairia.pop <- seppop (B_insularis) %>% lapply (pair.ia, sample = 1000)

##Testing linkage disequilibrium per pair

set.seed(999)

pairia.metapop <- pair.ia (B_insularis, sample = 1000)

set.seed(999)

pairia.pop <- seppop (B_insularis) %>% lapply (pair.ia, sample = 1000)

##Estimating Hardy-Weinberg Equilibrium per population

set.seed(999)

nanhwe.pop <- seppop (B_insularis) %>% lapply (hw.test, B = 1000)

nanhwe.mat <- sapply (nanhwe.pop, "[", i = TRUE, j = 4)

#STEP FOUR – GENETIC DIVERSITY AND INBREEDING COEFFCIENT PER KINSHIP INDEXES

B_insularis <- read.genepop ("B_insularis_clean_comoffspring.gen", ncode=3, quiet = FALSE)

#Accessing genetic diversity indexes

populations <- seppop (B_insularis)

set.seed (999)

sapply (populations,locus_table)

set.seed (999)

private_alleles (B_insularis)

set.seed (999)

allelic.richness (B_insularis)

set.seed (999)

summary (B_insularis)

#Inbreeding coefficient per kinship index

set.seed (999)

inbred_coef <- sapply (populations, inbreeding, res.type = "estimate")

Fis_Bar <- sapply (inbred_coef, mean)

Fis_Bar

set.seed(999)

boot.ppfis (dat = B_insularis, nboot =1000, quant = c (0.0025,0.998), diploid = TRUE, dig=4)

#STEP FIVE – POPULATIONAL DIFFERENTIATION INDEXES

B_insularis_off <- read.genepop ("testedapc.gen", ncode = 3, quiet = FALSE)

#Acessing the population differentiation indexes

set.seed (999)

testefst <- as.loci (B_insularis_off)

fsttab <- Fst (testefst)

fsttab

fstat (B_insularis_off)

set.seed (999)

pop_stru <- diff_stats (B_insularis_off)

#Estimating confidence intervals

set.seed (999)

bs_reps <- chao_bootstrap (B_insularis_off, nreps = 999)

summarise_bootstrap (bs_reps, D_jost)

summarise_bootstrap (bs_reps, Gst_Hedrick)

summarise_bootstrap (bs_reps, Gst_Nei)

#STEP SIX – Analysis of Molecular Variance

B_insularis_off <- read.genepop ("testedapc.gen", ncode = 3, quiet = FALSE)

#Gathering the hierarchic information

strata <- read.table ("B_insularis_strutpop.txt", header = TRUE, sep = "\t")

strata (B_insularis_off) <- other (B_insularis_off) <- strata [-1]

#AMOVA

set.seed(999)

amova.result <- poppr.amova (B_insularis_off, ~Pop, filter = FALSE)

set.seed(999)

amovasignificance <- randtest (amova.result, nrepet = 999)

amovasignificance

#STEP SEVEN – DISCRIMINANT ANALYSIS OF PRINCIPAL COMPONENTS

B_insularis_off <- read.genepop ("testedapc.gen", ncode=3, quiet = FALSE)

#Infering the number of genetic clusters

set.seed(999)

grp <- find.clusters (B_insularis_off, max.n.clust = 10)

30 #number of PCs

5 #number of clusters

#DAPC analysis

set.seed (999)

dapc1<- dapc (B_insularis_off, grp$grp)

25 #number of PCs

3 #number of discriminants

set.seed (999)

optim.a.score (dapc1)

set.seed (999)

dapc2 <- dapc (B_insularis_off, grp$grp, n.pca = 6)

3 #number of discriminants

myCol <- c ("darkblue","darkgrey","darkgreen","orange", "red")

compoplot (dapc2, col.pal = myCol, border = "black", show.lab = TRUE, lab = rownames ("B_insularis_off"), txt.leg = paste ("Cluster", 1:5), posi = "topleft", cleg=.7)
