# Supplementary material for: Genetic variability, management, and conservation implications of the critically endangered Brazilian pitviper Bothrops insularis
Source: Ecol Evol. 2020 Oct 3;10(23):12870–82. doi: 10.1002/ece3.6838 (PMC7713924; doi:10.1002/ece3.6838)
Supplement: Supplementary file 4 — AppendixS4 [file ECE3-10-12870-s004.docx]

# Appendix S4

**Article title:** Genetic variability, management, and conservation implications of the critically endangered Brazilian pitviper *Bothrops insularis*

**Journal name:** Ecology and Evolution

**Author names:** Igor Salles de Oliveira, Taís Machado, Karina Banci, Selma Maria Almeida-Santos, and Maria José de J. Silva.

**Corresponding author:** Maria José de J. Silva.

**Affiliation:** Laboratório de Ecologia e Evolução – Instituto Butantan, Av. Dr. Vital Brazil, 1500 – 05503-000 – São Paulo, SP, Brazil.

**E-mail:** mariajose.silva@butantan.gov.br

***
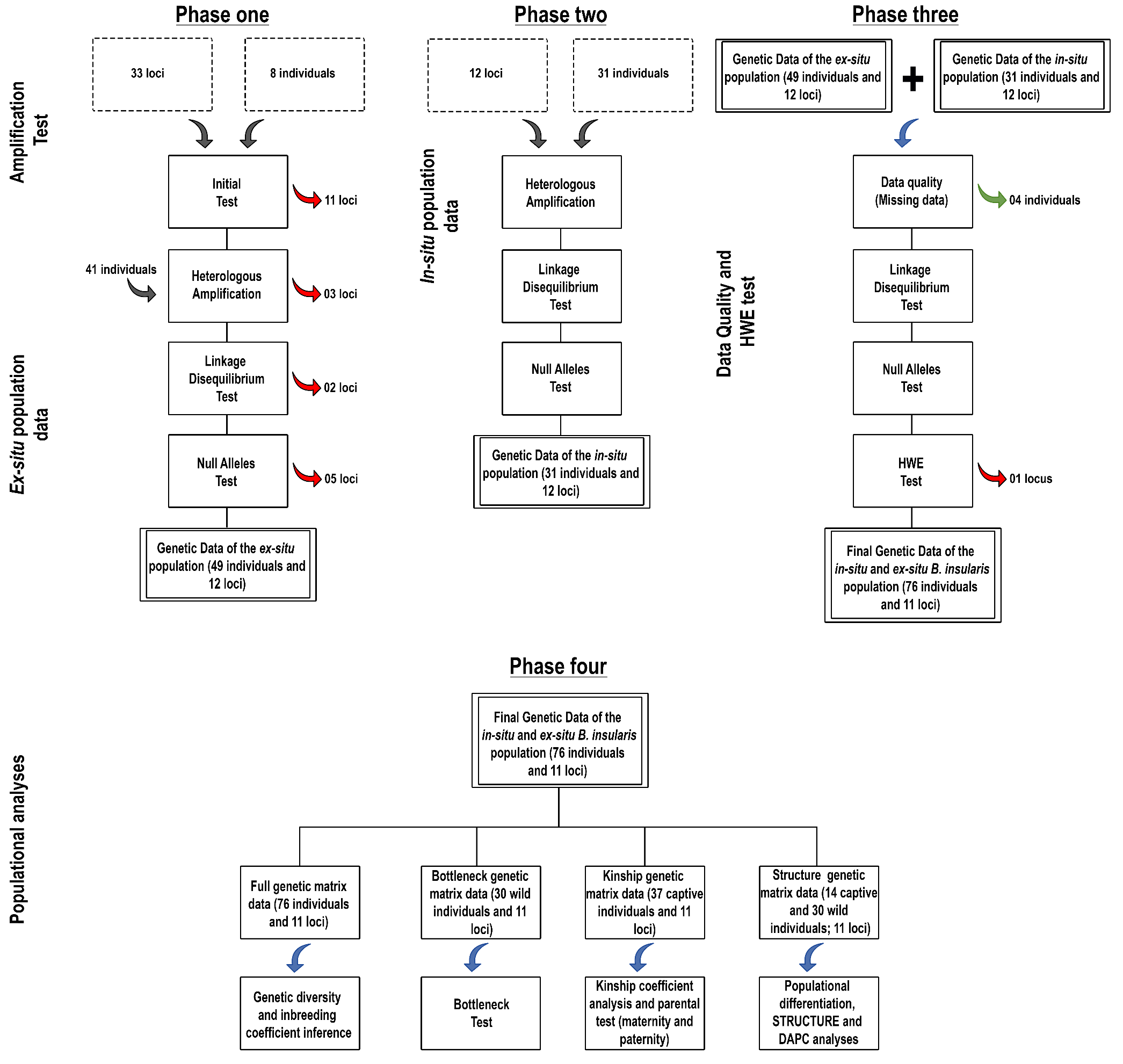
­***

**Fig. S1 –** Simplified scheme of the methodology and results developed in this study. Phase one consisted of the amplification tests using heterologous microsatellites and the tests performed to obtain the first genetic population data of the captive (ex-situ) population. Phase two consisted of the amplification using the informative 12 microsatellites of the Phase one and the tests performed to obtain the first genetic data of the wild (in-situ) population. Phase three was based on the tests performed to avoid biases in the subsequent populational analyses using the genetic data obtained in the Phases one and two. Phase four was composed of the following populational analyses: (i) genetic diversity and inbreeding inference using the genetic data obtained in the Phase three; (ii) bottleneck test using the wild population representatives; (iii) kinship coefficient and parental tests using captive individuals; and genetic structure analyses using 44 individuals (14 ex-situ and 30 in-situ) with no relationship recognized.
