# Supplementary material for: Genetic variability, management, and conservation implications of the critically endangered Brazilian pitviper Bothrops insularis
Source: Ecol Evol. 2020 Oct 3;10(23):12870–82. doi: 10.1002/ece3.6838 (PMC7713924; doi:10.1002/ece3.6838)
Supplement: Supplementary file 5 — AppendixS5 [file ECE3-10-12870-s005.docx]

# Appendix S5

**Article title:** Genetic variability, management, and conservation implications of the critically endangered Brazilian pitviper *Bothrops insularis*

**Journal name:** Ecology and evolution

**Author names:** Igor Salles de Oliveira, Taís Machado, Karina Banci, Selma Maria Almeida-Santos, and Maria José de J. Silva.

**Corresponding author:** Maria José de J. Silva.

**Affiliation:** Laboratório de Ecologia e Evolução – Instituto Butantan, Av. Dr. Vital Brazil, 1500 – 05503-000 – São Paulo, SP, Brazil.

**E-mail:** mariajose.silva@butantan.gov.br

**Table S2 –** Microsatellite genotype of each representative of the Bothrops insularis ex-situ (ID) and in-situ (KB) populations.

| Sample ID | Bmar_076 | Bpau_002 | Bpau_014 | Bpau_059 | Bpau_083 | Bpau_130 | Bmat_010 | Bmat_049 | Bmat_060 | Bmat_070 | Bmat_080 | Bmat_106 |
| --- | --- | --- | --- | --- | --- | --- | --- | --- | --- | --- | --- | --- |
| ID0002 | 196/200 | 233/238 | 247/247 | 117/133 | 261/261 | 147/147 | 258/266 | 153/153 | 138/138 | 117/117 | 193/193 | 167/173 |
| ID0003 | 196/200 | 233/238 | - | 117/138 | 261/261 | 126/147 | 258/262 | 153/157 | - | 117/117 | 193/193 | 167/173 |
| ID0004 | 196/200 | 229/233 | 251/251 | 133/138 | 261/264 | 126/147 | 258/262 | 153/157 | 133/138 | 117/117 | 190/193 | 167/173 |
| ID0005 | 196/200 | 233/238 | 247/247 | 117/133 | 258/261 | 147/147 | 250/262 | 149/153 | 122/122 | 117/117 | 190/193 | 167/173 |
| ID0006 | 196/200 | 233/238 | 247/251 | 117/117 | 261/264 | 126/147 | - | - | 121/121 | 117/117 | 193/193 | 167/173 |
| ID0007 | 196/196 | 233/238 | - | 133/142 | 261/264 | 126/126 | - | 153/153 | - | 117/117 | 193/193 | 167/173 |
| ID0008 | 196/196 | 233/238 | 247/247 | 117/133 | 261/264 | 147/147 | 258/262 | 153/153 | 133/138 | 117/117 | 193/193 | 167/173 |
| ID0009 | 196/200 | 233/238 | 247/251 | 133/138 | 258/261 | 126/147 | 262/266 | 153/157 | 133/138 | 117/117 | 190/193 | 167/173 |
| ID0010 | 196/200 | 238/243 | 247/247 | 133/138 | 261/264 | 126/147 | 258/262 | 153/153 | 121/138 | 117/117 | 193/193 | 167/173 |
| ID0011 | 196/196 | 233/233 | 247/247 | 133/142 | 261/264 | 126/147 | 262/270 | 153/153 | 121/138 | 117/117 | 193/193 | 167/173 |
| ID0012 | 196/196 | 233/238 | 247/251 | 117/117 | 261/264 | 147/147 | 258/262 | 153/157 | 121/121 | 117/121 | 193/193 | 167/173 |
| ID0013 | 196/196 | 233/238 | 247/251 | 117/117 | 261/264 | 147/147 | 258/262 | 153/157 | 121/121 | 117/121 | 193/193 | 167/176 |
| ID0016 | 196/200 | - | - | 117/133 | 261/261 | 147/147 | - | 153/157 | - | 117/117 | 193/193 | 167/173 |
| ID0017 | 196/196 | - | - | 97/138 | 261/264 | 126/147 | - | 153/153 | 121/121 | 117/117 | 193/193 | 167/167 |
| ID0018 | 196/200 | 233/238 | 247/251 | 133/138 | 261/264 | - | 258/262 | 153/157 | 129/138 | 117/117 | 193/193 | 167/173 |
| ID001F | 196/196 | 233/238 | 247/251 | 117/142 | 261/264 | 126/126 | 262/262 | 153/157 | 121/121 | - | - | 167/173 |
| ID002F | 196/200 | 233/238 | 247/251 | 117/117 | 261/264 | 126/147 | 262/270 | 153/157 | 121/129 | 117/121 | 193/193 | 167/173 |
| ID004F | 196/200 | - | 247/251 | 117/117 | 261/261 | 147/147 | 262/270 | 153/157 | 129/133 | 117/121 | 193/193 | 167/173 |
| ID005F | - | - | 247/247 | - | 261/261 | - | - | - | 138/138 | - | - | 167/167 |
| ID006F | 196/200 | - | 247/247 | 113/138 | 261/261 | 147/147 | 258/262 | 153/153 | 121/133 | 117/121 | 193/193 | 167/173 |
| ID009F | 196/200 | 233/238 | 247/247 | 117/133 | 261/264 | 147/147 | 262/266 | 153/153 | 121/133 | 117/117 | 193/193 | - |
| ID011F | 196/200 | 233/233 | 247/251 | 117/133 | 261/261 | 126/147 | 262/266 | 153/157 | 129/133 | 117/121 | 193/193 | 167/173 |
| ID013F | 196/200 | - | 247/247 | 117/138 | 261/264 | 126/126 | - | 153/153 | 121/121 | 117/117 | 193/193 | 167/173 |

**Sequel Table S2 –** Microsatellite genotype of each representative of the Bothrops insularis ex-situ (ID) and in-situ (KB) populations.

| Sample ID | Bmar_076 | Bpau_002 | Bpau_014 | Bpau_059 | Bpau_083 | Bpau_130 | Bmat_010 | Bmat_049 | Bmat_060 | Bmat_070 | Bmat_080 | Bmat_106 |
| --- | --- | --- | --- | --- | --- | --- | --- | --- | --- | --- | --- | --- |
| ID014F | 196/196 | 238/243 | - | 117/133 | 261/261 | 126/126 | 258/262 | 153/157 | - | 117/117 | - | 167/173 |
| ID015F | 196/200 | 233/238 | 247/251 | 117/138 | 261/264 | 126/147 | 258/262 | 153/157 | 121/129 | 117/117 | 193/193 | - |
| ID017F | 196/196 | 238/238 | 247/251 | 117/138 | 261/261 | - | 258/262 | 153/157 | 121/138 | 117/121 | 193/193 | 167/173 |
| ID021F | 196/200 | 233/238 | 247/251 | 117/138 | 261/261 | 147/147 | 258/262 | 153/157 | 121/133 | 117/121 | 193/193 | 167/173 |
| ID022F | 196/200 | 233/238 | 247/251 | 117/142 | 261/261 | 126/147 | 258/262 | 153/157 | 121/138 | 117/121 | 193/193 | 167/173 |
| ID024F | 196/196 | 233/233 | 247/251 | 117/133 | 261/264 | 147/147 | 258/262 | 153/157 | 121/138 | 117/121 | 193/193 | 167/173 |
| ID01FF | 196/200 | 233/238 | 251/251 | 133/138 | 261/264 | 126/126 | 262/266 | 153/157 | 133/138 | 117/117 | 190/193 | 167/173 |
| ID02FF | 196/200 | 233/238 | 247/251 | 133/133 | 261/264 | 147/147 | 258/262 | 153/157 | 133/138 | 117/117 | 193/193 | 167/173 |
| ID03FF | 196/200 | 233/238 | 247/251 | 133/138 | 261/264 | 126/147 | 262/266 | 153/157 | 133/138 | 117/117 | 193/193 | 167/173 |
| ID04FF | 196/200 | 233/238 | 247/251 | - | 261/264 | 147/147 | 258/262 | 153/157 | 133/138 | 117/117 | 193/193 | 167/173 |
| ID05FF | 196/200 | 233/238 | 247/251 | 133/138 | 261/264 | 126/126 | 262/266 | 153/157 | 133/138 | - | 193/193 | 167/173 |
| ID06FF | 196/200 | 238/238 | 247/247 | 133/133 | - | - | 258/262 | 153/153 | - | 117/117 | 193/193 | 167/173 |
| ID08FF | 196/196 | 233/238 | 247/247 | 133/133 | 261/278 | 147/147 | 258/262 | 153/153 | - | 117/121 | 193/193 | 167/173 |
| ID09FF | 196/200 | 233/238 | 247/247 | 117/133 | 264/278 | 147/147 | 262/262 | 153/153 | 133/138 | 117/117 | 193/193 | 167/173 |
| ID11FF | 196/200 | 233/238 | 247/247 | 117/117 | 261/261 | 126/147 | - | 153/153 | 121/121 | 117/117 | 193/193 | 167/173 |
| ID12FF | 196/200 | 233/238 | 247/247 | 117/138 | 261/264 | 147/147 | 258/262 | 153/153 | 121/129 | 117/121 | 193/193 | 167/173 |
| ID13FF | 196/200 | - | - | - | 261/264 | 147/147 | 258/262 | 153/157 | 121/138 | 117/117 | 193/193 | 167/173 |
| ID14FF | 196/196 | 233/238 | 247/251 | 117/133 | 261/261 | 147/147 | 258/262 | 153/157 | 121/129 | 117/121 | 193/193 | 167/173 |
| ID16FF | 196/196 | 233/238 | 247/251 | 117/138 | 261/261 | 126/147 | 262/266 | 153/157 | 138/138 | 117/117 | 193/193 | 167/173 |
| ID19FF | 196/196 | 233/238 | 247/247 | 117/138 | 261/261 | 126/147 | 262/266 | 153/153 | 138/138 | 117/117 | 193/193 | 167/173 |
| ID20FF | 196/196 | 233/238 | 247/247 | 133/138 | 261/261 | 126/147 | 262/266 | 153/153 | 133/133 | 117/117 | 193/193 | 167/173 |
| ID21FF | 196/196 | 233/233 | 247/251 | 133/138 | 261/261 | 126/147 | 262/266 | 153/157 | 129/133 | 117/117 | 193/193 | 167/173 |
| ID22FF | 196/200 | 233/238 | 247/251 | 117/138 | 261/261 | 126/147 | 258/262 | 153/157 | 133/133 | 117/117 | 193/193 | 167/173 |
| ID23FF | 196/196 | 233/238 | - | - | - | 126/147 | - | 153/153 | - | 117/117 | - | - |
| ID26FF | 196/196 | - | 247/251 | 117/117 | - | 126/147 | 262/266 | 153/157 | 121/138 | 121/121 | 193/193 | 167/173 |
| ID27FF | 196/200 | 233/233 | 247/247 | 117/117 | 261/261 | 126/147 | 262/266 | 153/153 | 121/138 | 117/121 | 193/193 | 167/173 |
| KB0001 | 200/200 | 238/238 | 247/247 | - | - | 147/147 | 262/266 | 153/153 | 121/121 | 113/117 | 193/193 | - |
| KB0002 | 196/200 | 233/238 | 251/251 | - | 261/264 | 126/147 | 262/262 | 157/157 | 121/133 | 113/117 | 193/193 | 167/173 |
| KB0003 | 196/200 | 238/238 | 247/247 | 138/138 | 261/264 | 126/147 | 262/266 | 153/153 | 133/137 | 113/117 | 193/193 | 167/173 |
| KB0004 | 196/200 | 238/238 | 247/251 | 133/138 | - | 126/147 | 262/262 | 153/157 | 121/137 | 113/117 | 193/193 | 167/173 |
| KB0005 | 196/200 | 233/238 | 247/247 | 117/138 | - | 126/147 | 262/266 | 153/153 | 121/137 | 113/117 | 193/193 | 167/173 |
| KB0006 | 200/200 | 233/238 | 247/247 | 133/133 | 261/264 | 147/147 | 262/270 | 153/153 | 121/137 | 113/117 | 193/193 | 167/173 |

**Sequel Table S2 –** Microsatellite genotype of each representative of the Bothrops insularis ex-situ (ID) and in-situ (KB) populations.

| Sample ID | Bmar_076 | Bpau_002 | Bpau_014 | Bpau_059 | Bpau_083 | Bpau_130 | Bmat_010 | Bmat_049 | Bmat_060 | Bmat_070 | Bmat_080 | Bmat_106 |
| --- | --- | --- | --- | --- | --- | --- | --- | --- | --- | --- | --- | --- |
| KB0007 | 196/200 | 233/238 | 247/247 | - | - | 147/147 | 262/262 | 153/153 | 125/133 | 117/121 | 207/207 | 167/173 |
| KB0008 | 196/196 | 233/233 | 247/251 | 117/133 | - | 147/147 | 262/262 | 153/157 | 121/137 | 113/117 | 193/193 | 167/173 |
| KB0009 | 196/200 | 233/238 | 238/247 | 133/138 | 258/261 | 126/147 | 262/262 | 153/153 | 129/129 | 113/117 | 204/207 | 167/173 |
| KB0010 | 196/196 | 233/238 | 247/247 | 133/138 | 261/261 | 126/147 | 262/262 | 153/153 | 121/129 | 113/117 | 193/193 | 167/173 |
| KB0011 | 196/200 | 233/238 | 251/251 | 117/133 | 261/264 | 147/147 | 266/266 | 157/157 | 121/129 | 113/117 | 193/193 | 167/173 |
| KB0012 | 196/196 | 233/238 | 243/247 | 117/117 | - | 147/147 | 262/266 | 153/153 | 133/137 | 113/117 | 193/193 | 167/173 |
| KB0013 | 196/200 | 233/238 | 243/247 | 138/138 | 261/261 | 147/147 | 262/266 | 153/153 | 133/137 | 113/117 | 193/193 | 167/173 |
| KB0014 | 196/196 | 233/233 | 247/247 | 117/133 | 261/261 | 126/147 | 258/262 | 153/153 | 121/129 | 109/113 | 193/193 | 167/173 |
| KB0015 | 196/200 | 233/238 | 243/247 | 133/138 | 261/264 | 147/147 | 262/262 | 153/153 | 121/121 | 113/117 | 193/193 | 167/173 |
| KB0016 | 196/200 | 233/248 | 247/251 | 133/138 | 261/261 | 147/147 | 262/266 | 153/157 | 133/137 | 117/121 | 193/193 | 167/173 |
| KB0017 | 196/196 | 233/238 | 247/247 | 142/142 | 261/261 | 147/147 | 262/262 | 153/153 | 125/133 | 113/117 | 193/193 | 167/173 |
| KB0018 | 196/200 | 233/238 | 247/247 | 138/138 | 261/261 | 126/147 | 262/262 | 153/153 | 121/121 | 113/117 | 193/193 | 167/173 |
| KB0019 | 196/200 | 233/238 | 247/247 | - | 261/261 | 147/147 | 258/266 | 153/153 | 133/137 | 113/117 | 193/193 | 167/173 |
| KB0020 | 196/200 | 233/238 | - | 117/138 | 261/261 | 126/147 | 262/262 | 153/153 | 121/137 | 113/117 | 193/193 | 167/173 |
| KB0021 | 200/200 | 233/238 | 247/251 | 117/138 | 264/264 | 126/147 | - | 153/157 | 121/121 | 113/117 | 193/193 | 167/173 |
| KB0022 | 200/200 | 233/233 | 247/247 | 117/138 | 261/261 | 147/147 | 262/266 | 153/153 | 121/121 | 113/117 | 193/193 | 167/173 |
| KB0023 | 196/200 | 238/248 | 247/247 | 117/133 | 261/261 | 147/147 | 262/262 | 153/153 | 121/121 | 117/121 | 193/193 | 167/173 |
| KB0024 | 196/200 | 233/233 | 247/247 | 133/138 | 261/264 | 147/147 | 266/270 | 153/153 | 125/133 | 113/117 | 193/193 | 167/173 |
| KB0025 | 200/200 | 233/238 | 247/251 | 117/133 | 261/261 | 147/147 | 262/266 | 157/157 | 121/133 | 113/117 | 193/193 | 167/173 |
| KB0026 | 196/200 | 233/238 | 243/247 | 117/133 | 261/261 | 126/147 | 258/266 | 153/153 | 133/137 | 113/117 | 193/193 | 167/173 |
| KB0027 | 200/200 | 233/238 | - | 117/133 | 261/264 | 147/147 | 258/266 | 153/153 | 125/133 | 113/117 | 193/193 | 167/173 |
| KB0028 | 200/200 | - | - | - | 261/264 | 126/147 | 262/262 | 153/157 | 121/121 | 113/117 | 193/193 | 167/173 |
| KB0029 | 196/200 | - | - | - | 261/264 | 147/147 | 258/262 | 153/157 | 121/121 | 113/117 | 193/193 | 167/173 |
| KB0030 | 196/200 | - | - | - | 261/261 | 147/147 | 262/262 | 153/157 | 133/133 | 113/117 | 193/193 | 167/173 |
| KB0031 | 196/200 | - | - | - | 258/261 | 147/147 | 262/266 | 153/157 | 133/137 | 113/117 | 193/193 | - |

(-) = Absence of amplification.
