# Supplementary material for: Genetic variability, management, and conservation implications of the critically endangered Brazilian pitviper Bothrops insularis
Source: Ecol Evol. 2020 Oct 3;10(23):12870–82. doi: 10.1002/ece3.6838 (PMC7713924; doi:10.1002/ece3.6838)
Supplement: Supplementary file 6 — AppendixS6 [file ECE3-10-12870-s006.docx]

# Appendix S6

**Article title:** Genetic variability, management, and conservation implications of the critically endangered Brazilian pitviper *Bothrops insularis*

**Journal name:** Ecology and Evolution

**Author names:** Igor Salles de Oliveira, Taís Machado, Karina Banci, Selma Maria Almeida-Santos, and Maria José de J. Silva.

**Corresponding author:** Maria José de J. Silva.

**Affiliation:** Laboratório de Ecologia e Evolução – Instituto Butantan, Av. Dr. Vital Brazil, 1500 – 05503-000 – São Paulo, SP, Brazil.

**E-mail:** mariajose.silva@butantan.gov.br

**Table S3 –** Relatedness value (R) obtained for each representative from Bothrops insularis ex-situ. Note that the table encompasses only the individuals that are currently alive (34). The mean kinship showed in the article for the ex situ population is calculated as the weighted average of the mean kinship from each individual.

| **Samples ID** | **ID0004** | **ID0006** | **ID0007** | **ID0008** | **ID0009** | **ID0010** | **ID0011** | **ID0012** | **ID0013** | **ID0017** | **ID0018** | **ID001F** | **ID002F** | **ID006F** | **ID009F** | **ID011F** | **ID014F** | **ID015F** | **ID017F** | **ID022F** | **ID01FF** | **ID02FF** | **ID03FF** | **ID08FF** | **ID09FF** | **ID11FF** | **ID13FF** | **ID14FF** | **ID16FF** | **ID19FF** | **ID20FF** | **ID22FF** | **ID26FF** | **ID27FF** |
| --- | --- | --- | --- | --- | --- | --- | --- | --- | --- | --- | --- | --- | --- | --- | --- | --- | --- | --- | --- | --- | --- | --- | --- | --- | --- | --- | --- | --- | --- | --- | --- | --- | --- | --- |
| **ID0004** | 1 |  |  |  |  |  |  |  |  |  |  |  |  |  |  |  |  |  |  |  |  |  |  |  |  |  |  |  |  |  |  |  |  |  |
| **ID0006** | 0 | 1 |  |  |  |  |  |  |  |  |  |  |  |  |  |  |  |  |  |  |  |  |  |  |  |  |  |  |  |  |  |  |  |  |
| **ID0007** | 0 | 0.06 | 1 |  |  |  |  |  |  |  |  |  |  |  |  |  |  |  |  |  |  |  |  |  |  |  |  |  |  |  |  |  |  |  |
| **ID0008** | 0.1 | 0 | 0.46 | 1 |  |  |  |  |  |  |  |  |  |  |  |  |  |  |  |  |  |  |  |  |  |  |  |  |  |  |  |  |  |  |
| **ID0009** | 0.48 | 0.14 | 0 | 0 | 1 |  |  |  |  |  |  |  |  |  |  |  |  |  |  |  |  |  |  |  |  |  |  |  |  |  |  |  |  |  |
| **ID0010** | 0.29 | 0.07 | 0.32 | 0.32 | 0.06 | 1 |  |  |  |  |  |  |  |  |  |  |  |  |  |  |  |  |  |  |  |  |  |  |  |  |  |  |  |  |
| **ID0011** | 0 | 0 | 0.71 | 0.4 | 0 | 0.32 | 1 |  |  |  |  |  |  |  |  |  |  |  |  |  |  |  |  |  |  |  |  |  |  |  |  |  |  |  |
| **ID0012** | 0 | 0.47 | 0.01 | 0.19 | 0 | 0 | 0 | 1 |  |  |  |  |  |  |  |  |  |  |  |  |  |  |  |  |  |  |  |  |  |  |  |  |  |  |
| **ID0013** | 0 | 0.47 | 0.01 | 0.19 | 0 | 0 | 0 | 1 | 1 |  |  |  |  |  |  |  |  |  |  |  |  |  |  |  |  |  |  |  |  |  |  |  |  |  |
| **ID0017** | 0 | 0.47 | 0.37 | 0.19 | 0 | 0.5 | 0.46 | 0.21 | 0.21 | 1 |  |  |  |  |  |  |  |  |  |  |  |  |  |  |  |  |  |  |  |  |  |  |  |  |
| **ID0018** | 0.39 | 0.47 | 0.06 | 0.06 | 0.34 | 0.33 | 0 | 0.28 | 0.28 | 0 | 1 |  |  |  |  |  |  |  |  |  |  |  |  |  |  |  |  |  |  |  |  |  |  |  |
| **ID001F** | 0 | 0.61 | 0.67 | 0 | 0 | 0 | 0.5 | 0.47 | 0.47 | 0.3 | 0.14 | 1 |  |  |  |  |  |  |  |  |  |  |  |  |  |  |  |  |  |  |  |  |  |  |
| **ID002F** | 0 | 0.76 | 0 | 0 | 0.02 | 0 | 0.14 | 0.48 | 0.48 | 0 | 0.25 | 0.5 | 1 |  |  |  |  |  |  |  |  |  |  |  |  |  |  |  |  |  |  |  |  |  |
| **ID006F** | 0 | 0 | 0 | 0.1 | 0 | 0.26 | 0 | 0.02 | 0.02 | 0 | 0 | 0 | 0 | 1 |  |  |  |  |  |  |  |  |  |  |  |  |  |  |  |  |  |  |  |  |
| **ID009F** | 0 | 0.5 | 0.19 | 0.54 | 0.03 | 0.08 | 0.02 | 0 | 0 | 0 | 0 | 0 | 0 | 0.31 | 1 |  |  |  |  |  |  |  |  |  |  |  |  |  |  |  |  |  |  |  |
| **ID011F** | 0 | 0 | 0 | 0 | 0.16 | 0 | 0 | 0 | 0 | 0 | 0.16 | 0 | 0.5 | 0 | 0.07 | 1 |  |  |  |  |  |  |  |  |  |  |  |  |  |  |  |  |  |  |
| **ID014F** | 0 | 0 | 0.29 | 0.2 | 0 | 0.5 | 0 | 0 | 0 | 0 | 0.03 | 0.19 | 0 | 0 | 0 | 0.04 | 1 |  |  |  |  |  |  |  |  |  |  |  |  |  |  |  |  |  |
| **ID015F** | 0.21 | 0.73 | 0 | 0 | 0.14 | 0.14 | 0 | 0.51 | 0.51 | 0.5 | 0.77 | 0.5 | 0.53 | 0 | 0 | 0.11 | 0 | 1 |  |  |  |  |  |  |  |  |  |  |  |  |  |  |  |  |
| **ID017F** | 0 | 0 | 0 | 0 | 0 | 0 | 0 | 0.56 | 0.56 | 0.07 | 0 | 0.48 | 0.02 | 0.3 | 0 | 0.09 | 0.38 | 0.2 | 1 |  |  |  |  |  |  |  |  |  |  |  |  |  |  |  |
| **ID022F** | 0 | 0.41 | 0.33 | 0 | 0.02 | 0.12 | 0.18 | 0.53 | 0.53 | 0 | 0.26 | 0.54 | 0.32 | 0.14 | 0 | 0.32 | 0 | 0.33 | 0.57 | 1 |  |  |  |  |  |  |  |  |  |  |  |  |  |  |
| **ID01FF** | 0.63 | 0 | 0.23 | 0.07 | 0.73 | 0.07 | 0 | 0 | 0 | 0 | 0.35 | 0.19 | 0 | 0 | 0.14 | 0.31 | 0.1 | 0.01 | 0 | 0 | 1 |  |  |  |  |  |  |  |  |  |  |  |  |  |
| **ID02FF** | 0.49 | 0.31 | 0 | 0.62 | 0.26 | 0.21 | 0 | 0.35 | 0.35 | 0 | 0.77 | 0.04 | 0.14 | 0 | 0.41 | 0 | 0 | 0.48 | 0 | 0.17 | 0.27 | 1 |  |  |  |  |  |  |  |  |  |  |  |  |
| **ID03FF** | 0.44 | 0.54 | 0.29 | 0.11 | 0.72 | 0.22 | 0 | 0 | 0 | 0 | 0.63 | 0.04 | 0.31 | 0 | 0.46 | 0.23 | 0 | 0.45 | 0 | 0.11 | 0.76 | 0.62 | 1 |  |  |  |  |  |  |  |  |  |  |  |
| **ID08FF** | 0 | 0 | 0 | 0.52 | 0 | 0.03 | 0.1 | 0.3 | 0.3 | 0 | 0 | 0 | 0 | 0.41 | 0.12 | 0 | 0 | 0 | 0.06 | 0.01 | 0 | 0.24 | 0 | 1 |  |  |  |  |  |  |  |  |  |  |
| **ID09FF** | 0 | 0 | 0 | 0.69 | 0.06 | 0.2 | 0.26 | 0 | 0 | 0 | 0 | 0 | 0 | 0.02 | 0.65 | 0 | 0 | 0 | 0 | 0 | 0.06 | 0.53 | 0.1 | 0.5 | 1 |  |  |  |  |  |  |  |  |  |
| **ID11FF** | 0 | 0.61 | 0 | 0 | 0 | 0.14 | 0.04 | 0.16 | 0.16 | 0.31 | 0 | 0.35 | 0.24 | 0.11 | 0.5 | 0 | 0.17 | 0.37 | 0.05 | 0.34 | 0 | 0 | 0 | 0 | 0.14 | 1 |  |  |  |  |  |  |  |  |
| **ID13FF** | 0.35 | 0.54 | 0 | 0.54 | 0 | 0.55 | 0.12 | 0.58 | 0.58 | 0.23 | 0.83 | 0 | 0.05 | 0.11 | 0.23 | 0 | 0.14 | 0.51 | 0.3 | 0.37 | 0.09 | 0.84 | 0.22 | 0.01 | 0.5 | 0.11 | 1 |  |  |  |  |  |  |  |
| **ID14FF** | 0 | 0 | 0 | 0.23 | 0 | 0 | 0 | 0.69 | 0.69 | 0 | 0.37 | 0 | 0.29 | 0.15 | 0 | 0.52 | 0.41 | 0.29 | 0.63 | 0.35 | 0 | 0.18 | 0 | 0.54 | 0 | 0 | 0.12 | 1 |  |  |  |  |  |  |
| **ID16FF** | 0 | 0.02 | 0 | 0 | 0.45 | 0 | 0 | 0 | 0 | 0 | 0.2 | 0 | 0 | 0 | 0 | 0.22 | 0.14 | 0.32 | 0.54 | 0.1 | 0.5 | 0 | 0.6 | 0 | 0 | 0 | 0 | 0.1 | 1 |  |  |  |  |  |
| **ID19FF** | 0 | 0 | 0.14 | 0.32 | 0.18 | 0.19 | 0.12 | 0 | 0 | 0.14 | 0 | 0 | 0 | 0 | 0.18 | 0.02 | 0 | 0.05 | 0.3 | 0 | 0.03 | 0 | 0.45 | 0 | 0.02 | 0.35 | 0 | 0 | 0.69 | 1 |  |  |  |  |
| **ID20FF** | 0 | 0 | 0.5 | 0.48 | 0.33 | 0.2 | 0.02 | 0 | 0 | 0.14 | 0.06 | 0 | 0 | 0.33 | 0.5 | 0.35 | 0.05 | 0 | 0 | 0 | 0.18 | 0.09 | 0.56 | 0.26 | 0.13 | 0.35 | 0 | 0 | 0.32 | 0.64 | 1 |  |  |  |
| **ID22FF** | 0.39 | 0.28 | 0 | 0.05 | 0.28 | 0 | 0 | 0.03 | 0.03 | 0 | 0.41 | 0 | 0.11 | 0.41 | 0 | 0.26 | 0.24 | 0.66 | 0.33 | 0.49 | 0.37 | 0.33 | 0.52 | 0 | 0 | 0.14 | 0.26 | 0.17 | 0.48 | 0.17 | 0.24 | 1 |  |  |
| **ID26FF** | 0 | 0.26 | 0 | 0 | 0.16 | 0 | 0.06 | 0.5 | 0.5 | 0 | 0 | 0.5 | 0.45 | 0 | 0 | 0.35 | 0 | 0 | 0.59 | 0.55 | 0 | 0 | 0.24 | 0 | 0 | 0.05 | 0.08 | 0.43 | 0.46 | 0.18 | 0.1 | 0 | 1 | . |
| **ID27FF** | 0 | 0.39 | 0 | 0 | 0 | 0.19 | 0.23 | 0.15 | 0.15 | 0 | 0 | 0 | 0.14 | 0.24 | 0.43 | 0.4 | 0 | 0 | 0.06 | 0.35 | 0 | 0 | 0.02 | 0 | 0 | 0.62 | 0.01 | 0 | 0.42 | 0.5 | 0.21 | 0 | 0.6 | 1 |
| **Mean** | 0.14 | 0.27 | 0.17 | 0.22 | 0.16 | 0.19 | 0.14 | 0.25 | 0.25 | 0.15 | 0.25 | 0.22 | 0.20 | 0.12 | 0.19 | 0.15 | 0.11 | 0.27 | 0.21 | 0.25 | 0.18 | 0.26 | 0.28 | 0.13 | 0.14 | 0.19 | 0.27 | 0.21 | 0.19 | 0.17 | 0.21 | 0.23 | 0.21 | 0.18 |
