# Supplementary material for: Genetic variability, management, and conservation implications of the critically endangered Brazilian pitviper Bothrops insularis
Source: Ecol Evol. 2020 Oct 3;10(23):12870–82. doi: 10.1002/ece3.6838 (PMC7713924; doi:10.1002/ece3.6838)
Supplement: Supplementary file 7 — AppendixS7 [file ECE3-10-12870-s007.docx]

# Appendix S7

**Article title:** Genetic variability, management, and conservation implications of the critically endangered Brazilian pitviper *Bothrops insularis*

**Journal name:** Ecology and Evolution

**Author names:** Igor Salles de Oliveira, Taís Machado, Karina Banci, Selma Maria Almeida-Santos, and Maria José de J. Silva.

**Corresponding author:** Maria José de J. Silva.

**Affiliation:** Laboratório de Ecologia e Evolução – Instituto Butantan, Av. Dr. Vital Brazil, 1500 – 05503-000 – São Paulo, SP, Brazil.

**E-mail:** mariajose.silva@butantan.gov.br

**
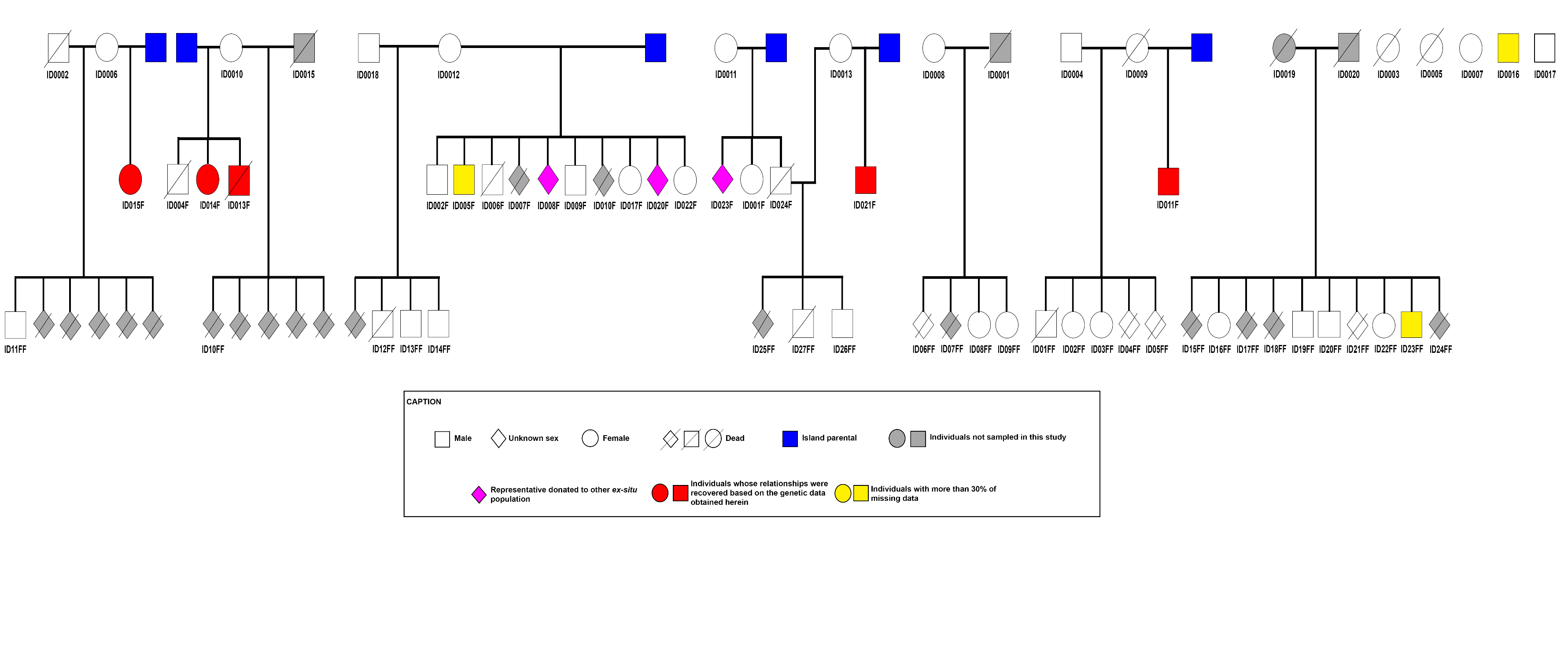
**

**Fig.S2 –** Pedigree reconstruction of the Bothrops insularis ex-situ population housed in the Laboratório de Ecologia e Evolução, Instituto Butantan, São Paulo State, Brazil. The relationships showed in the genogram were recovered based on the management data assigned until September 2019, and the genetic data provided herein. Blue individuals refer to island parental. Pink refers to individuals that were donated to other captive population. Gray refers to representatives from which we did not obtain samples. Yellow refers to individuals that had more than 30% missing genetic data. Red refers to offsprings for which the maternal relationship was recovered due to the genetic analysis of this study.
